# Immunogenicity and Safety of a Full-Dose Regimen of Cell Culture-Derived Quadrivalent Inactivated Influenza Vaccine in Children Aged 6–35 Months: Results from a Multinational Phase 3 Randomised Controlled Trial

**DOI:** 10.3390/vaccines14040341

**Published:** 2026-04-13

**Authors:** Yoonsun Yoon, Hye-Kyung Cho, Ki Hwan Kim, Su Eun Park, Yae-Jean Kim, Jina Lee, Hwang Min Kim, Nam Hee Kim, Dae Sun Jo, Eun Young Cho, Seon Hee Shin, Jong-Hyun Kim, Ji Hwa Ryu, Ho Keun Park, Yoonyeong Lee, Yun-Kyung Kim

**Affiliations:** 1 Department of Pediatrics, Korea University College of Medicine, Seoul 02841, Republic of Korea; yhappy815@korea.ac.kr; 2 Department of Pediatrics, Gachon University College of Medicine, Incheon 21565, Republic of Korea; 3 Department of Pediatrics, Incheon St. Mary’s Hospital, College of Medicine, The Catholic University of Korea, Seoul 21431, Republic of Korea; 4 Department of Pediatrics, Pusan National University College of Medicine, Yangsan 50612, Republic of Korea; 5 Department of Pediatrics, School of Medicine, Sungkyunkwan University, Seoul 06351, Republic of Korea; 6 Department of Pediatrics, University of Ulsan College of Medicine, Asan Medical Center, Seoul 05505, Republic of Korea; 7 Department of Pediatrics, Yonsei University, Wonju College of Medicine, Wonju 26426, Republic of Korea; 8 Department of Pediatrics, Inje University Ilsan Paik Hospital, Goyang 10380, Republic of Korea; 9 Department of Pediatrics, Jeonbuk National University Medical School, Jeonju 54907, Republic of Korea; 10 Department of Pediatrics, Chungnam National University Hospital, Daejeon 35015, Republic of Korea; 11 Department of Pediatric & Adolescent Medicine, Hallym University Dongtan Sacred Heart Hospital, Hwaseong 18450, Republic of Korea; 12 Department of Pediatrics, College of Medicine, The Catholic University of Korea, Seoul 06591, Republic of Korea; 13 Department of R&D, SK Bioscience, Seongnam 13494, Republic of Korea

**Keywords:** quadrivalent influenza vaccines, cell culture techniques, inactivated vaccine, seasonal influenza, immunogenicity, safety, children, clinical trial

## Abstract

**Background:** Influenza causes substantial morbidity in young children, particularly those aged 6–35 months. In this age group, optimisation of vaccine dose regimens remains important to ensure adequate immunogenicity while maintaining acceptable safety. This study evaluated the immunogenicity and safety of a full 0.5 mL dose of quadrivalent inactivated influenza vaccine (NBP607-QIV) in young children. **Methods:** This Phase 3, randomised, double-blind, active-controlled, multicentre study was conducted in Korea, Thailand, and Malaysia. Healthy children aged 6–35 months were randomised 2:1 to receive NBP607-QIV (0.5 mL) or control vaccine (0.25 mL). Immunogenicity was assessed using the haemagglutination inhibition assay. Primary endpoints were non-inferiority of NBP607-QIV versus Agrippal for seroconversion rate (SCR) and adjusted post-vaccination geometric mean titre (GMT) ratio against three shared strains. Immunogenicity against the additional B/Yamagata strain was evaluated according to Committee for Medicinal Products for Human Use (CHMP) criteria. Safety was assessed based on adverse events. **Results:** A total of 676 participants were randomised, and 675 were included in the safety set. Non-inferiority of NBP607-QIV versus control vaccine was demonstrated for SCR for all shared strains and for the adjusted GMT ratio for A/H1N1 and B/Victoria, but not for A/H3N2. Immunogenicity against the B/Yamagata strain met CHMP criteria for SCR and geometric mean ratio (GMR). Immunogenicity was consistent across prespecified subgroups, and the incidence of adverse events was comparable between groups, with no clinically meaningful safety concerns. **Conclusions:** NBP607-QIV administered at a 0.5 mL dose demonstrated acceptable immunogenicity and a safety profile comparable to that of a licensed trivalent influenza vaccine in children aged 6–35 months, supporting its use in this paediatric population.

## 1. Introduction

Influenza remains a major global public health concern, with a disproportionate burden observed among young children. Recent estimates from the World Health Organization (WHO) and national public health authorities indicate that seasonal influenza continues to cause substantial morbidity and mortality worldwide, particularly in children younger than 5 years of age [1,2]. Importantly, approximately 99% of influenza-associated deaths due to lower respiratory tract infections in this age group occur in developing countries, highlighting persistent global inequities in access to preventive strategies and healthcare resources. Among paediatric populations, children aged 6–35 months are especially vulnerable owing to immunological immaturity and an increased risk of influenza-related complications, including otitis media, lower respiratory tract infections, and hospitalisation [3,4].

Vaccination represents the most effective strategy for preventing influenza and its complications, and many countries, including the United States, have incorporated routine influenza vaccination for children aged ≥6 months into their national immunisation programmes [5]. Historically, inactivated influenza vaccines administered to children aged 6–35 months were administered at a reduced dose of 0.25 mL—half the standard adult dose—largely due to concerns regarding reactogenicity [6]. Advances in vaccine formulation, particularly the introduction of split-virus inactivated influenza vaccines, have improved safety profiles compared with earlier whole-virus vaccines [7,8], and accumulating clinical evidence has demonstrated that full-dose regimens can enhance immunogenicity while maintaining acceptable safety profiles in children aged 6–35 months [9,10]. Reflecting these findings, several regulatory authorities have approved full-dose inactivated influenza vaccines for this age group, and immunisation guidelines in North America, Europe, and parts of Asia have been updated accordingly [11,12,13].

NBP607-QIV (SKYCellflu^®^ Quadrivalent, SK bioscience, Seongnam, Republic of Korea) is a cell culture-derived, inactivated subunit quadrivalent influenza vaccine that has been evaluated in phase 3 clinical studies across multiple age groups. In a pivotal phase 3 trial in healthy adults aged ≥19 years, including elderly participants aged ≥60 years, NBP607-QIV demonstrated immunogenicity comparable to that of the trivalent NBP607 vaccine for the three shared strains and superior responses to the additional B strain; the vaccine met Committee for Medicinal Products for Human Use (CHMP) immunogenicity criteria for all four strains (A/H1N1, A/H3N2, B/Victoria, and B/Yamagata) and showed an acceptable safety and tolerability profile [14]. In a subsequent phase 3 trial in healthy children and adolescents aged 6 months to 18 years, NBP607-QIV administered at a 0.25 mL dose met CHMP criteria overall, although lower immune responses to influenza B strains were observed in participants younger than 3 years of age in both the NBP607-QIV and control groups [15].

Based on this evidence, NBP607-QIV received initial marketing authorisation for adult use in Korea in December 2015, followed by an extension of indication to children and adolescents aged ≥36 months in June 2016. In consideration of the reduced immunogenicity observed in children younger than 3 years and evolving regulatory expectations for this age group, a full 0.5 mL dose of NBP607-QIV was subsequently developed for evaluation in children aged 6–35 months. Accordingly, this phase 3, randomised, double-blind, active-controlled study evaluated the immunogenicity and safety of a 0.5 mL dose of NBP607-QIV in healthy children aged 6–35 months, with Agrippal^®^ (Novartis Vaccines and Diagnostics Srl, Siena, Italy), an egg-based inactivated influenza vaccine, used as the active comparator.

## 2. Materials and Methods

### 2.1. Ethics

This study was designed by SK bioscience (Seongnam, Republic of Korea) and conducted in accordance with the International Council for Harmonisation (ICH) Guidelines for Good Clinical Practice and the principles of the Declaration of Helsinki. The study protocol was reviewed and approved by the national regulatory authorities of Korea, Thailand, and Malaysia, as well as by the Institutional Review Board or Ethics Committee at each participating study centre. Written informed consent was obtained from the parent(s) or legally authorised representative(s) of all participants prior to the performance of any study-specific procedures. The full study protocol is not publicly available; however, key protocol elements are described in the Section 2 and the trial registration at ClinicalTrials.gov (NCT03704740).

### 2.2. Study Design and Participants

This was a randomised, double-blind, active-controlled, phase 3 clinical trial conducted at multiple centres across three countries (12 sites in Korea, five sites in Thailand, and three sites in Malaysia) during the 2018–2019 Northern Hemisphere and 2019 Southern Hemisphere influenza seasons. Eligible participants were healthy infants and toddlers aged 6–35 months. For participants aged 6 to 12 months, only those born after a normal gestational period (37 weeks or more) were included. Written informed consent was obtained from the parent(s) or legally authorised representative(s) of each participant following a full explanation of the study procedures. Children were excluded if they had immunodeficiency, malignancy, a history of vaccine-related hypersensitivity (including Guillain–Barré syndrome), or bleeding disorders contraindicating intramuscular injection. Participants with febrile illness (body temperature ≥38 °C) within 72 h prior to vaccination, or who had received immunosuppressive or immunomodulatory therapy within 12 weeks prior to screening, were not eligible, except for those using topical, nasal, or inhaled corticosteroids. Additional exclusion criteria included receipt of blood products or immunoglobulins within 24 weeks, any influenza vaccine within 24 weeks, or any other vaccine within 4 weeks before or after study vaccination. Individuals recently involved in other clinical trials, or with serious chronic or clinically significant medical or psychiatric conditions deemed unsuitable by the investigator, were not enrolled.

**Figure 1:**
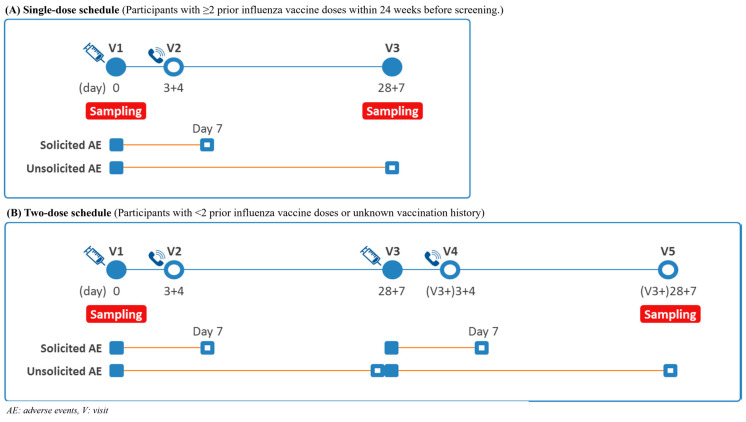
Study design and schedule.

Participants were randomised to receive a single dose of either NBP607-QIV or control vaccine. Children who had not received at least two prior doses of influenza vaccine within 24 weeks before screening, or whose influenza vaccination history could not be reliably documented, received a second dose of the assigned study vaccine according to the same treatment allocation. Blood samples for immunogenicity assessment were collected at baseline and 28 days after the final vaccination. The vaccination and blood sampling schedule is illustrated in Figure 1. There were no protocol amendments that affected the study objectives, endpoints, or statistical analyses.

### 2.3. Randomisation and Masking

Eligible participants were randomised in a 2:1 ratio to receive NBP607-QIV or the control vaccine using a central Interactive Web Response System (IWRS). Randomisation was stratified by age group (6–17 months and 18–35 months) and study site to minimise potential bias. The randomisation schedule was generated by an independent statistician not otherwise involved in the conduct of the study. To maintain double-blind conditions, only designated unblinded pharmacists, vaccinators, and unblinded study monitors were responsible for tasks related to the management and administration of the investigational vaccine. All other study personnel, participants, and parents or legally authorised representatives remained blinded to treatment allocation throughout the study.

### 2.4. Vaccines

The investigational vaccine, NBP607-QIV, is a quadrivalent, inactivated subunit influenza vaccine manufactured by SK bioscience. It is a cell culture-derived vaccine produced using Madin–Darby canine kidney (MDCK) cells. Each 0.5 mL prefilled syringe contains 15 µg of haemagglutinin (HA) from each of the four influenza virus strains recommended by the World Health Organization (WHO) for the 2018–2019 Northern Hemisphere and 2019 Southern Hemisphere influenza seasons: A/Michigan/45/2015 (H1N1)pdm09-like virus, A/Singapore/INFIMH-16-0019/2016 (H3N2)-like virus, B/Colorado/06/2017-like virus (B/Victoria lineage), and B/Phuket/3073/2013-like virus (B/Yamagata lineage).

The control vaccine, Agrippal^®^, is a licensed trivalent, egg-derived, inactivated subunit influenza vaccine. Each 0.5 mL of prefilled syringe of control vaccine contains 15 µg of HA from the same three influenza strains as NBP607-QIV, with the exception of the B/Yamagata lineage strain. In accordance with the approved indication for children aged 6–35 months, a 0.25 mL dose of control vaccine was administered to participants assigned to the control group.

### 2.5. Objectives

The primary immunogenicity objective was to demonstrate the non-inferiority of NBP607-QIV compared with control vaccine with respect to immune responses against A/H1N1, A/H3N2, and B/Victoria strains, as assessed by adjusted post-vaccination geometric mean titres (GMTs) and seroconversion rates (SCRs) measured using the haemagglutination inhibition (HI) assay. For the B/Yamagata strain, immunogenicity was evaluated by SCR and geometric mean ratio (GMR) in accordance with the criteria established by the CHMP. The secondary immunogenicity objective was to evaluate immune responses 28 days after the final dose of NBP607-QIV according to the CHMP criteria. The safety objective was to compare the safety profile and reactogenicity of NBP607-QIV with those of control vaccine.

### 2.6. Immunogenicity Assessment

Serological analyses were conducted at SK bioscience Life Science Research Laboratory (Seongnam, Republic of Korea). Immunogenicity was evaluated using the HI assay, a well-established and widely accepted method for assessing antibody responses to influenza vaccines. The HI assay quantified serum antibodies directed against the haemagglutinin antigens of the A/H1N1, A/H3N2, B/Victoria, and B/Yamagata strains included in the study vaccine. Assays were conducted using cell-derived haemagglutinin antigens and chicken erythrocytes in accordance with standardised procedures.

HI assay results were evaluated using four predefined immunogenicity endpoints: adjusted post-vaccination GMT (post-vaccination GMT adjusted for pre-vaccination titre), seroprotection rate (the proportion of participants achieving a post-vaccination HI titre ≥1:40), seroconversion rate (the proportion of participants with a post-vaccination HI titre increase from <1:10 to ≥1:40 or a ≥4-fold increase from a pre-vaccination titre ≥1:10), and geometric mean ratio (the fold increase in GMT from baseline to post-vaccination).

### 2.7. Safety Assessment

Following administration of either 0.5 mL of NBP607-QIV or 0.25 mL of control vaccine, participants were observed on site for at least 30 min to monitor for immediate adverse reactions. Parents or legally authorised representatives were provided with a participant diary and instructed to record all adverse events (AEs) occurring from the day of vaccination through 28 days after the final study vaccination.

Safety assessments included the incidence of solicited and unsolicited AEs and serious adverse events (SAEs). Solicited local AEs (pain/tenderness, erythema/redness, and induration/swelling) and solicited systemic AEs (fever, whining/irritability, and sleepiness/fatigue) occurring within 7 days after the final vaccination were recorded in the diary. Unsolicited AEs were collected for 21 days following vaccination, and SAEs were recorded throughout the study period. All AEs were coded according to the Medical Dictionary for Regulatory Activities (MedDRA), version 22.0, using system organ class (SOC) and preferred term (PT).

Investigators assessed the severity, seriousness, and causal relationship of all reported AEs. AE severity was graded in accordance with the US Food and Drug Administration (FDA) guidance, Toxicity Grading Scale for Healthy Adult and Adolescent Volunteers Enrolled in Preventive Vaccine Clinical Trials, and the Ministry of Food and Drug Safety (MFDS) guideline, Severity Assessment of Adverse Events in Vaccine Clinical Trials.

### 2.8. Statistical Analysis

All randomised participants were included in the intention-to-treat (ITT) population for demographic analyses, and all participants who received at least one dose of the study vaccine were included in the safety set. Immunogenicity analyses were performed on the per-protocol set (PPS), comprising participants who completed the study without major protocol deviations.

The primary objective was to demonstrate the non-inferiority of NBP607-QIV compared with control vaccine with respect to HI antibody responses against the three shared influenza strains (A/H1N1, A/H3N2, and B/Victoria). Non-inferiority was established if the upper bound of the two-sided 95% confidence interval (CI) for the adjusted post-vaccination GMT ratio (Agrippal/NBP607-QIV) did not exceed 1.5 and if the upper bound of the 95% CI for the difference in seroconversion rate (SCR; Agrippal—NBP607-QIV) did not exceed 10%. Post-vaccination GMTs were analysed using analysis of covariance (ANCOVA) with pre-vaccination titres included as covariates. For the B/Yamagata strain, which was included only in NBP607-QIV, seroprotection rate (SPR), SCR, and GMR were assessed against the CHMP criteria (lower bounds of the 95% CI: SPR ≥70%, SCR ≥40%, and GMR ≥2.5).

Secondary immunogenicity analyses assessed whether the CHMP criteria were met for A/H1N1, A/H3N2, and B/Victoria strains and whether the lower bound of the 95% CI for SPR met the CHMP threshold (≥70%) for the B/Yamagata strain. Group comparisons for SPR and SCR were performed using the chi-square or Fisher’s exact test, while differences in GMR were analysed using the *t*-test. Consistency across countries was evaluated by comparing SCR differences and GMT ratios within each country using the same non-inferiority margins (10% for SCR and 1.5 for the GMT ratio).

Predefined subgroup analyses were conducted for immunogenicity by age (6–17 vs. 18–35 months), sex, country, baseline HI titre (<1:10 vs. ≥1:10), and vaccination schedule (one-dose vs. two-dose) and for safety by country and vaccination schedule, using the same statistical methods as the primary analyses. In dose-specific safety analyses, AEs and adverse drug reactions (ADRs) occurring after the first dose were summarised for all participants in the one-dose group, while for the two-dose group, only events occurring after the second dose were included. Adverse events that began after the first dose and persisted beyond the second were attributed to the one-dose group based on the onset time. The number and percentage of participants experiencing each AE and ADR, with corresponding 95% CIs for incidence rates, were summarised, and between-group differences were assessed using chi-square or Fisher’s exact tests, with exact 95% CIs presented when appropriate. All analyses were performed using observed data only, with no imputation of missing values.

Sample size calculations were based on demonstrating non-inferiority in post-vaccination GMT and SCR for A/H1N1, A/H3N2, and B/Victoria strains. Based on previous phase 3 data for NBP607-QIV (0.5 mL) and Agrippal (0.25 mL), a minimum of 450 participants in the NBP607-QIV group and 225 participants in the control group were planned, with at least 15% of participants recruited in Korea to ensure adequate regional representation.

The statistical analysis plan was prospectively defined but is not publicly available. Individual de-identified participant data, statistical code, and other study materials are likewise not publicly available. No interim analyses were planned or conducted, and no stopping guidelines were prespecified.

## 3. Results

### 3.1. Study Participants

**Figure 2:**
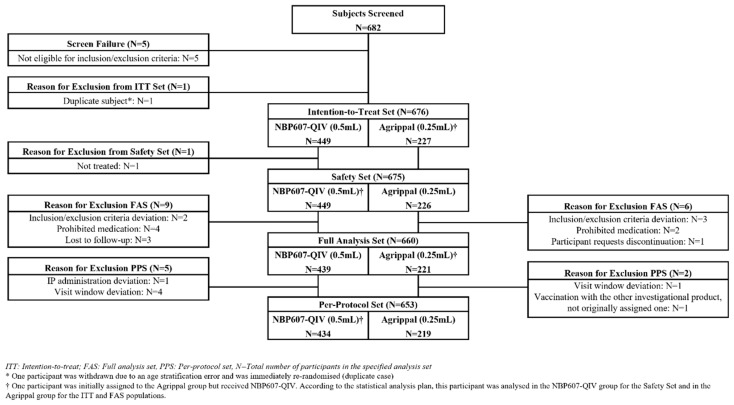
Flow diagram of study participants.

Between October 2018 and July 2019, a total of 682 participants were screened, of whom 676 eligible participants were enrolled and randomised to receive either NBP607-QIV (n = 449) or Agrippal (n = 227). Of these, 675 participants received at least one dose of the study vaccine and were included in the safety set, with the exception of one who did not receive vaccination and was therefore excluded from the safety analysis. Among participants who received one or two doses of the study vaccine, 653 participants (434 in the NBP607-QIV group and 219 in the control vaccine group) were included in the PPS. Participants were excluded from the PPS due to deviations from inclusion or exclusion criteria, major protocol deviations, receipt of prohibited concomitant therapies, loss to follow-up, or withdrawal of consent. Participant disposition throughout the study is summarised in Figure 2.

Baseline demographic characteristics, including age, sex, country, body weight, and height, were comparable between the two treatment groups. Based on prior influenza vaccination history, a total of 509 participants (340 in the NBP607-QIV group and 169 in the control vaccine group) received two doses of the assigned study vaccine. A summary of baseline demographic characteristics is presented in Table 1.

### 3.2. Immunogenicity

Post-vaccination SCR differences and adjusted GMT ratios, as assessed by the HI assay, are summarised in Table 2. Additional immunogenicity analyses, including detailed results for primary and secondary endpoints, are provided in Supplementary Tables S1–S3 and Figures S1–S15. HI assays were performed for each vaccine strain, reflecting the quadrivalent composition of NBP607-QIV. For the three strains shared by both vaccines (A/H1N1, A/H3N2, and B/Victoria), between-group differences in SCR (Agrippal − NBP607-QIV) were −1.71% (95% CI: −7.28, 3.85) for A/H1N1, 1.94% (95% CI: −3.17, 7.05) for A/H3N2, and −8.46% (95% CI: −16.53, −0.39) for B/Victoria. For all three strains, the upper bound of the 95% CI for the SCR difference did not exceed the predefined non-inferiority margin of 10%, thereby demonstrating non-inferiority of NBP607-QIV with respect to SCR.

Adjusted GMT ratios (Agrippal/NBP607-QIV) were 1.09 (95% CI: 0.95, 1.27) for A/H1N1, 1.31 (95% CI: 1.13, 1.52) for A/H3N2, and 0.93 (95% CI: 0.78, 1.10) for B/Victoria. Non-inferiority based on adjusted GMT ratio was demonstrated for the A/H1N1 and B/Victoria strains, as the upper bounds of the 95% CIs did not exceed the predefined margin of 1.5. However, the non-inferiority criterion was not met for the A/H3N2 strain, as the upper bound of the 95% CI marginally exceeded the prespecified threshold.

For the B/Yamagata strain, which was included only in NBP607-QIV, the SCR was 57.83% (95% CI: 53.19, 62.48) and the GMR was 6.11 (95% CI: 5.54, 6.73). Both endpoints met the CHMP immunogenicity criteria, with the lower bounds of the 95% CIs exceeding the predefined thresholds of 40% for SCR and 2.5 for GMR. Post-vaccination immunogenicity assessed by the HI assay was further evaluated against the predefined CHMP criteria for SPR, SCR, and GMR for all vaccine strains, as summarised in Table 3. In the NBP607-QIV group, both SPR and SCR for the A/H1N1 and A/H3N2 strains met the CHMP criteria with wide margins, whereas the lower bounds of the 95% CIs for SPR did not meet the CHMP threshold for the B/Victoria or B/Yamagata strains. In contrast, SCR and GMR met the CHMP criteria for all evaluated strains.

A similar pattern was observed in the control vaccine group, in which the CHMP criterion for SPR was not met for the B/Victoria strain. When comparing immunogenicity between vaccine groups, SPR and SCR for the B/Victoria strain were significantly higher in the NBP607-QIV group than in the control vaccine group.

In terms of individual distributions, both vaccines showed increases in HI titres from baseline to post-vaccination across all strains, with largely overlapping post-vaccination distributions between groups. Further detailed HI titre distributions are presented in Supplementary Figures S8–S11. For the B/Yamagata strain, an upward shift in titres was observed in the NBP607-QIV group, whereas responses in the control vaccine group were comparatively limited.

Subgroup analyses were performed to further evaluate immunogenicity according to age (6–17 months and 18–35 months), sex, pre-vaccination titre (<1:10 or ≥1:10), country (Korea, Thailand, and Malaysia), and vaccination regimen (one-dose or two-dose). No clinically meaningful differences in immune response patterns were observed between age groups or between sexes, and no consistent trends were identified according to vaccination regimen or baseline antibody titre. Immunogenicity against the A/H1N1, A/H3N2, B/Victoria, and B/Yamagata strains was consistent across participating countries, with no notable differences or trends in between-group SCR differences or GMRs, and with non-inferiority and CHMP criteria met consistently within each country. Detailed subgroup-specific results are presented in Supplementary Figures S12–S15.

### 3.3. Safety

Reported AEs are summarised in Table 4. Although the overall incidence of AEs was numerically higher in the control vaccine group, no statistically significant differences were observed between the two groups in the proportions of participants experiencing solicited local AEs (*p* = 0.8366), solicited systemic AEs (*p* = 0.8634), unsolicited AEs (*p* = 0.6376), or SAEs (*p* = 0.0851).

Among unsolicited AEs and SAEs classified by SOC, infections and infestations were the most frequently reported category in both groups. A total of 19 SAEs were reported within 28 days after vaccination. Of these, one SAE in the NBP607-QIV group—acute pharyngitis—was assessed as having a causal relationship with the study vaccine. The participant was hospitalised for two days and fully recovered without sequelae.

Most reported AEs were of grade 1 or 2 severity. One participant in the NBP607-QIV group experienced a grade 4 solicited systemic AE, characterised by a high fever exceeding 40 °C, which occurred in association with the aforementioned acute pharyngitis. Overall, the safety profiles were comparable between the two treatment groups. Detailed safety outcomes are summarised in Supplementary Tables S4–S8.

## 4. Discussion

This phase 3, randomised, double-blind study in children aged 6–35 months demonstrated that the immunogenicity of NBP607-QIV was non-inferior to that of a licensed trivalent influenza vaccine, Agrippal^®^, for two of the shared strains (A/H1N1 and B/Victoria) and that immune responses against the additional B/Yamagata strain met the CHMP criteria. The reactogenicity and overall safety profile of NBP607-QIV were comparable to those of control vaccine, indicating that inclusion of the B/Yamagata strain did not adversely affect vaccine safety.

Although non-inferiority based on the GMT ratio was not demonstrated for the A/H3N2 strain, non-inferiority was achieved for SCR across all three shared strains. The marginal failure to meet the GMT-based non-inferiority criterion for A/H3N2 should be interpreted in the context of the well-documented antigenic variability of H3N2 viruses, which has been associated with reduced and more variable immune responses across multiple influenza vaccine platforms. Consistent with previous reports, immune responses to A/H3N2 remain challenging to optimise, and the findings of the present study are in line with the broader literature [16,17].

In the secondary immunogenicity analyses based on the CHMP criteria, the lower bounds of the 95% CIs for SPR did not meet the predefined thresholds for the B/Victoria and B/Yamagata strains. This pattern has been observed in prior studies of licensed influenza vaccines and reflects the generally lower serological responses to influenza B strains compared with A strains in young children. [15,18,19]. Notably, despite this limitation, NBP607-QIV demonstrated higher immune responses to the B/Victoria strain than the comparator vaccine, suggesting a relative advantage in inducing B-lineage immunity.

The findings related to the B/Yamagata strain should be interpreted in light of recent epidemiological changes. Global circulation of the B/Yamagata lineage has markedly declined since the COVID-19 pandemic, and a transition toward trivalent influenza vaccine formulations has been observed. Nevertheless, inclusion of this strain reflects the standard of care at the time of trial design and provides a comprehensive assessment of vaccine immunogenicity.

Taken together, the immunogenicity data indicate that all four vaccine strains induced immune responses of clinical relevance, with robust and consistent responses observed for A/H1N1 and B/Victoria, acceptable responses for A/H3N2 despite its intrinsic antigenic variability, and meaningful immunogenicity against the additional B/Yamagata strain. These findings support the overall immunogenic potential of NBP607-QIV across all included strains in young children.

The immunogenicity findings of this study are consistent with those reported in a pivotal phase 3 study of Flucelvax^®^ (Seqirus, Maidenhead, UK), another cell culture-derived influenza vaccine evaluated in paediatric populations. In a pivotal phase 3 study, non-inferiority of GMT ratios and SCRs was not demonstrated for the H3N2 strain in children aged 4–9 years, and the CHMP criterion for seroprotection against influenza B strains was not met when compared with Fluvirin, a conventional egg-derived trivalent influenza vaccine [20]. Despite these strain-specific limitations, Flucelvax^®^ was approved by both the US FDA) and European Medicines Agency (EMA), underscoring that such findings are not uncommon in paediatric influenza vaccine studies and should be interpreted within an appropriate regulatory and clinical context [21,22].

In this context, the present findings are also consistent with evidence supporting the use of cell culture-derived influenza vaccines. Compared with egg-based vaccines, cell-based platforms avoid egg-adaptive changes in haemagglutinin and may therefore better preserve antigenic characteristics of circulating strains. This feature may contribute to improved immune responses, particularly for strains with greater antigenic variability [23,24]. In addition, the use of cell-derived antigens and an egg-free manufacturing process offers potential advantages in terms of product consistency and tolerability [25]. Together, these considerations support the continued evaluation of cell culture-based influenza vaccines in paediatric populations.

While administration of a 0.5 mL dose of NBP607-QIV was generally well tolerated and elicited appropriate immune responses in the paediatric population studied, several limitations of the present study should be acknowledged. First, immunogenicity comparisons between NBP607-QIV and control vaccine were based solely on the HI assay. Although HI remains a well-established and widely used method for assessing antibody responses to influenza vaccines, accumulating evidence indicates that viral microneutralisation assays can provide complementary information, particularly for strains characterised by greater antigenic variability [26,27]. In line with this, regulatory guidelines from major authorities, including the FDA and EMA, acknowledge the value of incorporating viral microneutralisation as a supplementary immunogenicity assessment [28,29,30]. Further evaluation using complementary assays would therefore be valuable to better characterise the immune responses elicited by NBP607-QIV.

Second, while the present study provides robust immunogenicity and safety data, it was not designed to directly assess clinical vaccine effectiveness. Given that the CHMP has highlighted the importance of demonstrating vaccine-induced protection and immune memory in children aged 6–36 months, further studies evaluating vaccine effectiveness in larger paediatric populations, potentially across multiple influenza seasons, may be warranted to more fully characterise the clinical impact of NBP607-QIV in this age group [30].

In conclusion, this phase 3 study demonstrates that NBP607-QIV, a quadrivalent cell culture-derived influenza vaccine, exhibits a favourable immunogenicity and safety profile that is comparable to that of Agrippal in children aged 6–35 months. These findings support the use of a 0.5 mL dose of NBP607-QIV in this paediatric age group.

## Figures and Tables

**Table 1:** Demographic and baseline characteristics.

	NBP607-QIV (0.5 mL) (N = 449)	Agrippal (0.25 mL) (N = 227)	Total (N = 676)
**Age (months)**			
n	449	227	676
Mean (SD)	23.92 (6.91)	24.01 (6.78)	23.95 (6.87)
Median	25.00	25.00	25.00
Min, Max	6.00, 35.00	6.00, 35.00	6.00, 35.00
*p*-value ^a^			0.8675 [t]
**Age group,** **N (%)**			
6~17 months	78 (17.37)	41 (18.06)	119 (17.60)
18~35 months	371 (82.63)	186 (81.94)	557 (82.40)
*p*-value ^b^			0.8240 [c]
**Country, N (%)**			
Korea	62 (13.81)	33 (14.54)	95 (14.05)
Thailand	292 (65.03)	144 (63.44)	436 (64.50)
Malaysia	95 (21.16)	50 (22.03)	145 (21.45)
*p*-value ^b^			0.9187 [c]
**Gender, N (%)**			
Male	227 (50.56)	111 (48.90)	338 (50.00)
Female	222 (49.44)	116 (51.10)	338 (50.00)
*p*-value ^b^			0.6839 [c]
**Dose regimen, N (%)**			
1-dose	109 (24.28)	58 (25.55)	167 (24.70)
2-dose	340 (75.72)	169 (74.45)	509 (75.30)
*p*-value ^b^			0.7167 [c]
**Influenza vaccination history, N (%)**			
None	327 (72.83)	164 (72.25)	491 (72.63)
Once	12 (2.67)	5 (2.20)	17 (2.51)
Twice and more	110 (24.50)	58 (25.55)	168 (24.85)
Unknown	0 (000)	0 (0.00)	0 (0.00)
*p*-value ^b^			0.9019 [c]

N = number, SD = standard deviation, Min = minimum, Max = maximum. ^a^ Testing for differences between treatment groups (two-sample *t*-test [t]). ^b^ Testing for differences between treatment groups (chi-square test [c]). Note: The denominator of the percentage is the number of subjects in each group. Note: Category ‘Unknown’ is excluded from the test for influenza vaccination history. Age (months) = difference in months between informed consent date and birth date. If the informed consent date was before the birth date, then 1 month was subtracted. If the subject received two or more doses of influenza vaccine until 24 weeks prior to screening or if such vaccination history was not confirmed, they received two doses, whereas those who did not meet this requirement received one dose.

**Table 2:** Immunogenicity by haemagglutination inhibition assay (primary endpoints).

	NBP607-QIV (0.5 mL) (N = 434)	Agrippal (0.25 mL) (N = 219)
**[** **A/H1N1]**		
**Pre-vaccination GMT (SD)**	16.39 (5.92)	21.75 (6.62)
95% confidence interval	[13.86, 19.39]	[16.91, 27.98]
**Post-vaccination GMT (SD)**	220.20 (3.95)	272.73 (4.99)
95% confidence interval	[193.44, 250.65]	[220.19, 337.82]
**Adjusted post-vaccination GMT (SE)**	229.70 (1.05)	251.45 (1.06)
95% confidence interval	[210.64, 250.50]	[222.90, 283.66]
GMR (Agrippal/NBP607-QIV) [95% CI] ^a^		1.09 [0.95, 1.27]
**Seroconversion rate, N (%)**	380 (87.56)	188 (85.84)
95% confidence interval	[84.45, 90.66]	[81.23, 90.46]
Difference % (Agrippal—NBP607-QIV) [95% CI]		−1.71 [−7.28, 3.85]
**[A/H3N2]**		
**Pre-vaccination GMT (SD)**	18.21 (5.77)	14.10 (4.95)
95% confidence interval	[15.44, 21.49]	[11.39, 17.44]
**Post-vaccination GMT (SD)**	218.64 (4.41)	239.90 (3.97)
95% confidence interval	[190.08, 251.48]	[199.65, 288.27]
**Adjusted post-vaccination GMT (SE)**	202.74 (1.05)	265.45 (1.06)
95% confidence interval	[185.86, 221.15]	[234.80, 300.11]
GMR (Agrippal/NBP607-QIV) [95% CI] ^a^		1.31 [1.13, 1.52]
**Seroconversion rate, N (%)**	380 (87.56)	196 (89.50)
95% confidence interval	[84.45, 90.66]	[85.44, 93.56]
Difference % (Agrippal—NBP607-QIV) [95% CI]		1.94 [−3.17, 7.05]
**[B/Victoria]**		
**Pre-vaccination GMT (SD)**	5.40 (1.45)	5.28 (1.24)
95% confidence interval	[5.22, 5.59]	[5.14, 5.44]
**Post-vaccination GMT (SD)**	26.42 (2.96)	24.14 (3.00)
95% confidence interval	[23.85, 29.27]	[20.85, 27.94]
**Adjusted post-vaccination GMT (SE)**	26.40 (1.05)	24.45 (1.07)
95% confidence interval	[23.93, 29.12]	[21.30, 28.06]
GMR (Agrippal/NBP607-QIV) [95% CI] ^a^		0.93 [0.78, 1.10]
**Seroconversion rate, N (%)**	223 (51.38)	94 (42.92)
95% confidence interval	[46.68, 56.08]	[36.37, 49.48]
Difference % (Agrippal—NBP607-QIV) [95% CI]		−8.46 [−16.53, −0.39]
**[B/Yamagata]**		
**Pre-vaccination GMT (SD)**	6.08 (1.74)	5.88 (1.58)
95% confidence interval	[5.77, 6.40]	[5.53, 6.24]
**Post-vaccination GMT (SD)**	37.10 (3.13)	6.74 (1.87)
95% confidence interval	[33.32, 41.32]	[6.20, 7.33]
**GMR (post/pre-vaccination) (SD)**	6.11 (2.79)	1.15 (1.49)
95% confidence interval	[5.54, 6.73]	[1.09, 1.21]
**Seroconversion rate, N (%)**	251 (57.83)	4 (1.83)
95% confidence interval	[53.19, 62.48]	[0.05, 3.60]

ANCOVA = analysis of covariance, UCL = upper confidence limit, LCL = lower confidence limit, GMT = geometric mean titre, GMR = geometric mean ratio, SD = standard deviation, SE = standard error, CI = confidence interval. ^a^ Testing for the ratio between treatment groups (ANCOVA model with treatment group as a factor and log pre-GMT as a covariate). Note: The denominator of the percentage is the number of subjects in the column. Seroconversion rate: proportion of subjects who have a post-vaccination HI titre of ≥1:40 for subjects with a pre-vaccination HI titre of <1:10 or a fourfold increase in post-vaccination HI titre for subjects with a pre-vaccination HI titre of ≥1:10. The confidence interval of GMT is calculated using the t-distribution, and the confidence interval of the seroconversion rate is calculated using the Wald method.

**Table 3:** Immunogenicity by haemagglutination inhibition assay according to CHMP criteria (secondary endpoints).

	NBP607-QIV (0.5 mL) (N = 434)	Agrippal (0.25 mL) (N = 219)
**[A/H1N1]**		
**Seroprotection rate,** **N (%)**	413 (95.16)	204 (93.15)
95% confidence interval	[93.14, 97.18]	[89.81, 96.50]
*p*-value ^a^		0.2879 [c]
**Seroconversion rate,** **N (%)**	380 (87.56)	188 (85.84)
95% confidence interval	[84.45, 90.66]	[81.23, 90.46]
*p*-value ^a^		0.5391 [c]
**GMR (post/pre-vaccination) (SD)**	13.43 (3.27)	12.54 (3.05)
95% confidence interval	[12.01, 15.02]	[10.81, 14.55]
*p*-value ^b^		0.4750
**[A/H3N2]**		
**Seroprotection rate,** **N (%)**	416 (95.85)	207 (94.52)
95% confidence interval	[93.98, 97.73]	[91.51, 97.53]
*p*-value ^a^		0.4427 [c]
**Seroconversion rate,** **N (%)**	380 (87.56)	196 (89.50)
95% confidence interval	[84.45, 90.66]	[85.44, 93.56]
*p*-value ^a^		0.4680 [c]
**GMR (post/pre-vaccination) (SD)**	12.00 (2.94)	17.02 (3.15)
95% confidence interval	[10.84, 13.29]	[14.61, 19.83]
*p*-value ^b^		0.0001
**[B/Victoria]**		
**Seroprotection rate,** **N (%)**	228 (52.53)	94 (42.92)
95% confidence interval	[47.84, 57.23]	[36.37, 49.48]
*p*-value ^a^		0.0204 [c]
**Seroconversion rate,** **N (%)**	223 (51.38)	94 (42.92)
95% confidence interval	[46.68, 56.08]	[36.37, 49.48]
*p*-value ^a^		0.0411 [c]
**GMR (post/pre-vaccination) (SD)**	4.89 (2.82)	4.57 (2.87)
95% confidence interval	[4.44, 5.39]	[3.97, 5.25]
*p*-value ^b^		0.4279
**[B/Yamagata]**		
**Seroprotection rate,** **N (%)**	259 (59.68)	11 (5.02)
95% confidence interval	[55.06, 64.29]	[2.13, 7.92]
*p*-value ^a^		<0.0001
**Seroconversion rate,** **N (%)**	251 (57.83)	4 (1.83)
95% confidence interval	[53.19, 62.48]	[0.05, 3.60]
*p*-value ^a^		<0.0001

LCL = lower confidence limit, GMR = geometric mean ratio. ^a^ Testing for differences between treatment groups (chi-square test [c]). ^b^ Testing for differences between treatment groups (two-sample *t*-test). Note: The denominator of the percentage is the number of subjects in the column. GMR is displayed as a geometric mean (geometric standard deviation). GMR = anti-logarithm [mean of logarithm(POST/PRE)] (PRE = original value before IP administration, POST = original value after IP administration). Seroprotection rate: proportion of subjects whose post-vaccination HI titre increased to ≥1:40. Seroconversion rate: the proportion of subjects who had a post-vaccination HI titre of ≥1:40 for subjects with a pre-vaccination HI titre of <1:10 or a fourfold increase in post-vaccination HI titre for subjects with a pre-vaccination HI titre of ≥1:10. The confidence interval of the seroconversion rate is calculated using the Wald method.

**Table 4:** Summary of overall adverse events.

	NBP607-QIV (0.5 mL) (N = 449)	Agrippal (0.25 mL) (N = 226)	Total (N = 675)	*p*-Value ^a^
**Total AEs,** **N (%)** **[case]**	314 (69.93) [927]	164 (72.57) [512]	478 (70.81) [1439]	0.4776 [c]
**Subjects with Solicited Local AEs,** **N (%) [case]**	187 (41.65) [336]	96 (42.48) [193]	283 (41.93) [529]	0.8366 [c]
Pain/Tenderness	96 (21.38) [114]	50 (22.12) [63]	146 (21.63) [177]	0.8249 [c]
Erythema/Redness	126 (28.06) [164]	68 (30.09) [93]	194 (28.74) [257]	0.5830 [c]
Induration/Swelling	52 (11.58) [58]	31 (13.72) [37]	83 (12.30) [95]	0.4253 [c]
**Subjects with Solicited Systemic AEs,** N **(%) [case]**	136 (30.29) [246]	67 (29.65) [123]	203 (30.07) [369]	0.8634 [c]
Fever	53 (11.80) [57]	28 (12.39) [28]	81 (12.00) [85]	0.8252 [c]
Whining/(Irritation)	84 (18.71) [103]	49 (21.68) [55]	133 (19.70) [158]	0.3594 [c]
Sleepiness/(Feeling drained)	72 (16.04) [86]	37(16.37) [40]	109 (16.15) [126]	0.9108 [c]
**Subjects with Unsolicited AEs,** N **(%) [case]**	202 (44.99) [345]	106 (46.90) [196]	308 (45.63) [541]	0.6376 [c]
**Subjects with Serious AEs,** N **(%) [case]**	8 (1.78) [8]	9 (3.98) [11]	17 (2.52) [19]	0.0851 [c]

AEs = adverse events. ^a^ Testing for differences between treatment groups (chi-square test [c]). Note: The denominator of the percentage is the number of subjects in each group.

## Data Availability

The data supporting the findings of this study are available from the corresponding author upon reasonable request, subject to applicable ethical and privacy restrictions.

## Supplementary Materials

The following are available online at https://www.mdpi.com/article/10.3390/vaccines14040341/s1, Table S1: Immunogenicity Assessment by HI Assay (Primary Endpoints)—Per Protocol Set; Table S2: Immunogenicity Assessment by HI Assay (Secondary Endpoints CHMP Criteria)—Per Protocol Set; Table S3: Immunogenicity Assessment by HI Assay (Secondary Endpoints_Consistency Among Countries—Per Protocol Set; Table S4: Overall Adverse Events—Safety Set; Table S5: Solicited Local Adverse Events—Safety Set; Table S6: Solicited Systemic Adverse Events—Safety Set; Table S7: Unsolicited Adverse Events—Safety Set; Table S8: Serious Adverse Events by System Organ Class / Preferred Term—Safety Set; Figure S1: Ratio of Adjusted post-vaccination GMTs (GMTAgrippal/GMTNBP607-QIV) by HI Assay [A/H1N1, A/H3N2, B/Victoria]—Per Protocol Set; Figure S2: Difference of Seroconversion Rates between Treatment Groups by HI Assay [A/H1N1, A/H3N2, B/Victoria]—Per Protocol Set; Figure S3: GMR (post/pre-vaccination of NBP607-QIV) by HI Assay [B/Yamagata]—Per Protocol Set; Figure S4: Seroconversion Rate post-vaccination of NBP607-QIV by HI Assay [B/Yamagata]—Per Protocol Set; Figure S5: Seroprotection Rates post-vaccination of NBP607-QIV/Agrippal by HI Assay [A/H1N1, A/H3N2, B/Victoria, B/Yamagata]—Per Protocol Set; Figure S6: Seroconversion Rates post-vaccination of NBP607-QIV/Agrippal by HI Assay [A/H1N1, A/H3N2, B/Victoria]—Per Protocol Set; Figure S7: GMRs (post/pre-vaccination of NBP607-QIV/Agrippal) by HI Assay [A/H1N1, A/H3N3, B/Victoria]—Per Protocol Set; Figure S8: Pre- and post-vaccination HI titre distributions [A/H1N1—Per Protocol Set; Figure S9: Pre- and post-vaccination HI titre distributions [A/H3N2]—Per Protocol Set; Figure S10: Pre- and post-vaccination HI titre distributions [B/Victoria]—Per Protocol Set; Figure S11: Pre- and post-vaccination HI titre distributions [B/Yamagata]—Per Protocol Set; Figure S12: Age-stratified distributions of HI titres post-vaccination [A/H1N1]—Per Protocol Set; Figure S13: Age-stratified distributions of HI titres post-vaccination [A/H3N2]—Per Protocol Set; Figure S14: Age-stratified distributions of HI titres post-vaccination [B/Victoria]—Per Protocol Set; Figure S15: Age-stratified distributions of HI titres post-vaccination [B/Yamagata]—Per Protocol Set.
