# Supplementary material for: Immunogenicity and Safety of a Full-Dose Regimen of Cell Culture-Derived Quadrivalent Inactivated Influenza Vaccine in Children Aged 6–35 Months: Results from a Multinational Phase 3 Randomised Controlled Trial
Source: Vaccines (Basel). 2026 Apr 13;14(4):341. doi: 10.3390/vaccines14040341 (PMC13119535; doi:10.3390/vaccines14040341)

Supplementary Materials

Supplement to: Yoon Y, Cho HY, Kim KH, et al. Immunogenicity and Safety of a Full-Dose Regimen of Cell Culture-Derived Quadrivalent Inactivated Influenza Vaccine in Children Aged 6–35 Months: Results from a Multinational Phase 3 Randomized Controlled Trial

Table of Contents

I. Supplementary Tables

Table S1 Immunogenicity Assessment by HI Assay (Primary Endpoints) – Per Protocol Set

Table S2 Immunogenicity Assessment by HI Assay (Secondary Endpoints_CHMP Criteria)– Per Protocol Set

Table S3 Immunogenicity Assessment by HI Assay (Secondary Endpoints_Consistency Among Countries – Per Protocol Set

Table S4 Overall Adverse Events – Safety Set

Table S5 Solicited Local Adverse Events – Safety Set

Table S6 Solicited Systemic Adverse Events – Safety Set

Table S7 Unsolicited Adverse Events – Safety Set

Table S8 Serious Adverse Events by System Organ Class / Preferred Term – Safety Set

II. Supplementary Figures

Figure S1 Ratio of Adjusted post-vaccination GMTs ($GMT_{Agrippal}/GMT_{NBP607\text{-}QIV}$) by HI Assay [A/H1N1, A/H3N2, B/Victoria] – Per Protocol Set

Figure S2 Difference of Seroconversion Rates between Treatment Groups by HI Assay [A/H1N1, A/H3N2, B/Victoria] – Per Protocol Set

Figure S3 GMR (post/pre-vaccination of NBP607-QIV) by HI Assay [B/Yamagata] – Per Protocol Set

Figure S4 Seroconversion Rate post-vaccination of NBP607-QIV by HI Assay [B/Yamagata] – Per Protocol Set

Figure S5 Seroprotection Rates post-vaccination of NBP607-QIV/Agrippal by HI Assay [A/H1N1, A/H3N2, B/Victoria, B/Yamagata] – Per Protocol Set

Figure S6 Seroconversion Rates post-vaccination of NBP607-QIV/Agrippal by HI Assay [A/H1N1, A/H3N2, B/Victoria] – Per Protocol Set

Figure S7 GMRs (post/pre-vaccination of NBP607-QIV/Agrippal) by HI Assay [A/H1N1, A/H3N3, B/Victoria] – Per Protocol Set

Figure S8 Pre- and post-vaccination HI titre distributions [A/H1N1 – Per Protocol Set

Figure S9 Pre- and post-vaccination HI titre distributions [A/H3N2] – Per Protocol Set

Figure S10 Pre- and post-vaccination HI titre distributions [B/Victoria] – Per Protocol Set

Figure S11 Pre- and post-vaccination HI titre distributions [B/Yamagata] – Per Protocol Set

Figure S12 Age-stratified distributions of HI titres post-vaccination [A/H1N1] – Per Protocol Set

Figure S13 Age-stratified distributions of HI titres post-vaccination [A/H3N2] – Per Protocol Set

Figure S14 Age-stratified distributions of HI titres post-vaccination [B/Victoria] – Per Protocol Set

Figure S15 Age-stratified distributions of HI titres post-vaccination [B/Yamagata] – Per Protocol Set

I. Supplementary Tables

Table S1 Immunogenicity Assessment by HI Assay (Primary Endpoints) – Per Protocol Set

Page 1 of 4

	NBP607-QIV (0.5mL) (N=434)	Agrippal (0.25mL) (N=219)
**[Influenza Strain: A/H1N1]**		
**Pre-vaccination GMT (SD)**	16.39(5.92)	21.75(6.62)
95% Confidence Interval	[13.86, 19.39]	[16.91, 27.98]
**Post-vaccination GMT (SD)**	220.20(3.95)	272.73(4.99)
95% Confidence Interval	[193.44, 250.65]	[220.19, 337.82]
**Adjusted post-vaccination GMT (SE)**	229.70(1.05)	251.45(1.06)
95% Confidence Interval	[210.64, 250.50]	[222.90, 283.66]
GMR (Agrippal/NBP607-QIV) [95% CI] [1]		1.09 [0.95, 1.27]
UCL ≤ 1.5?		Yes
**Seroconversion rate, n(%)**	380(87.56)	188(85.84)
95% Confidence Interval	[84.45, 90.66]	[81.23, 90.46]
Difference % (Agrippal - NBP607-QIV) [95% CI]		-1.71 [-7.28, 3.85]
UCL ≤ 10%?		Yes

ANCOVA = analysis of covariance, UCL = upper confidence limit, LCL = lower confidence limit, GMT = geometric mean titer, GMR = geometric mean ratio, SD = standard deviation, SE = standard error, CI = confidence interval.
[1] Testing for ratio between treatment groups (ANCOVA model with treatment group as a factor and log pre-GMT as a covariate).
Note: Denominator of percentage is the number of subjects in the column.
Seroconversion rate: proportion of subjects who post-vaccination HI titer of ≥1:40 for subjects with pre-vaccination HI titer of <1:10 or four-fold increase in post-vaccination HI titer for subjects with pre-vaccination HI titer of ≥1:10.
Confidence interval of GMT is calculated using t-distribution and Confidence Interval of Seroconversion rate is calculated using Wald method.

	NBP607-QIV (0.5mL) (N=434)	Agrippal (0.25mL) (N=219)
**[Influenza Strain: A/H3N2]**		
**Pre-vaccination GMT (SD)**	18.21(5.77)	14.10(4.95)
95% Confidence Interval	[15.44, 21.49]	[11.39, 17.44]
**Post-vaccination GMT (SD)**	218.64(4.41)	239.90(3.97)
95% Confidence Interval	[190.08, 251.48]	[199.65, 288.27]
**Adjusted post-vaccination GMT (SE)**	202.74(1.05)	265.45(1.06)
95% Confidence Interval	[185.86, 221.15]	[234.80, 300.11]
GMR (Agrippal/NBP607-QIV) [95% CI] [1]		1.31 [1.13, 1.52]
UCL ≤ 1.5?		No
**Seroconversion rate, n(%)**	380(87.56)	196(89.50)
95% Confidence Interval	[84.45, 90.66]	[85.44, 93.56]
Difference % (Agrippal - NBP607-QIV) [95% CI]		1.94 [-3.17, 7.05]
UCL ≤ 10%?		Yes

ANCOVA = analysis of covariance, UCL = upper confidence limit, LCL = lower confidence limit, GMT = geometric mean titer, GMR = geometric mean ratio, SD = standard deviation, SE = standard error, CI = confidence interval.
[1] Testing for ratio between treatment groups (ANCOVA model with treatment group as a factor and log pre-GMT as a covariate).
Note: Denominator of percentage is the number of subjects in the column.
Seroconversion rate: proportion of subjects who post-vaccination HI titer of ≥1:40 for subjects with pre-vaccination HI titer of <1:10 or four-fold increase in post-vaccination HI titer for subjects with pre-vaccination HI titer of ≥1:10.
Confidence interval of GMT is calculated using t-distribution and Confidence Interval of Seroconversion rate is calculated using Wald method.

	NBP607-QIV (0.5mL) (N=434)	Agrippal (0.25mL) (N=219)
**[Influenza Strain: B/Victoria]**		
**Pre-vaccination GMT (SD)**	5.40(1.45)	5.28(1.24)
95% Confidence Interval	[5.22, 5.59]	[5.14, 5.44]
**Post-vaccination GMT (SD)**	26.42(2.96)	24.14(3.00)
95% Confidence Interval	[23.85, 29.27]	[20.85, 27.94]
**Adjusted post-vaccination GMT (SE)**	26.40(1.05)	24.45(1.07)
95% Confidence Interval	[23.93, 29.12]	[21.30, 28.06]
GMR (Agrippal/NBP607-QIV) [95% CI] [1]		0.93 [0.78, 1.10]
UCL ≤ 1.5?		Yes
**Seroconversion rate, n(%)**	223(51.38)	94(42.92)
95% Confidence Interval	[46.68, 56.08]	[36.37, 49.48]
Difference % (Agrippal - NBP607-QIV) [95% CI]		-8.46 [-16.53, -0.39]
UCL ≤ 10%?		Yes

LCL = lower confidence limit, GMR = geometric mean ratio.
[1] Testing for difference between treatment groups (chi-square test (c) or Fisher's exact test (f)).
[2] Testing for difference between treatment groups (two sample t-test).
Note: Denominator of percentage is the number of subjects in the column. GMR are displayed as geometric mean (geometric standard deviation).
GMR = anti-logarithm[mean of logarithm(POST/PRE)] (PRE=original value before IP administration, POST= original value after IP administration)
Seroprotection rate: proportion of subjects whose post-vaccination HI titer increased to ≥1:40.
Seroconversion rate: proportion of subjects who post-vaccination HI titer of ≥1:40 for subjects with pre-vaccination HI titer of <1:10 or four-fold increase in post-vaccination HI titer for subjects with pre-vaccination HI titer of ≥1:10.

	NBP607-QIV (0.5mL) (N=434)	Agrippal (0.25mL) (N=219)
**[Influenza Strain: B/Yamagata]**		
**Pre-vaccination GMT (SD)**	6.08(1.74)	5.88(1.58)
95% Confidence Interval	[5.77, 6.40]	[5.53, 6.24]
**Post-vaccination GMT (SD)**	37.10(3.13)	6.74(1.87)
95% Confidence Interval	[33.32, 41.32]	[6.20, 7.33]
**GMR (post/pre-vaccination) (SD)**	6.11(2.79)	1.15(1.49)
95% Confidence Interval	[5.54, 6.73]	[1.09, 1.21]
LCL ≥ 2.5?	Yes	No
**Seroconversion rate, n(%)**	251(57.83)	4(1.83)
95% Confidence Interval	[53.19, 62.48]	[0.05, 3.60]
LCL ≥ 40%?	Yes	No

LCL = lower confidence limit, GMR = geometric mean ratio.
[1] Testing for difference between treatment groups (chi-square test (c) or Fisher's exact test (f)).
[2] Testing for difference between treatment groups (two sample t-test).
Note: Denominator of percentage is the number of subjects in the column. GMR are displayed as geometric mean (geometric standard deviation).
GMR = anti-logarithm[mean of logarithm(POST/PRE)] (PRE=original value before IP administration, POST= original value after IP administration)
Seroprotection rate: proportion of subjects whose post-vaccination HI titer increased to ≥1:40.
Seroconversion rate: proportion of subjects who post-vaccination HI titer of ≥1:40 for subjects with pre-vaccination HI titer of <1:10 or four-fold increase in post-vaccination HI titer for subjects with pre-vaccination HI titer of ≥1:10.

Table S2 Immunogenicity Assessment by HI Assay (Secondary Endpoints_CHMP Criteria)– Per Protocol Set

	NBP607-QIV (0.5mL) (N=434)	Agrippal (0.25mL) (N=219)
**[Influenza Strain: A/H1N1]**		
**Seroprotection rate, n(%)**	413(95.16)	204(93.15)
95% Confidence Interval	[93.14, 97.18]	[89.81, 96.50]
LCL ≥ 70%?	Yes	Yes
P-value [1]		0.2879 (c)
**Seroconversion rate, n(%)**	380(87.56)	188(85.84)
95% Confidence Interval	[84.45, 90.66]	[81.23, 90.46]
LCL ≥ 40%?	Yes	Yes
P-value [1]		0.5391 (c)
**GMR (post/pre-vaccination) (SD)**	13.43(3.27)	12.54(3.05)
95% Confidence Interval	[12.01, 15.02]	[10.81, 14.55]
LCL ≥ 2.5?	Yes	Yes
P-value [2]		0.4750

LCL = lower confidence limit, GMR = geometric mean ratio.
[1] Testing for difference between treatment groups (chi-square test (c) or Fisher's exact test (f)).
[2] Testing for difference between treatment groups (two sample t-test).
Note: Denominator of percentage is the number of subjects in the column. GMR are displayed as geometric mean (geometric standard deviation).
GMR = anti-logarithm[mean of logarithm(POST/PRE)] (PRE=original value before IP administration, POST= original value after IP administration)
Seroprotection rate: proportion of subjects whose post-vaccination HI titer increased to ≥1:40.
Seroconversion rate: proportion of subjects who post-vaccination HI titer of ≥1:40 for subjects with pre-vaccination HI titer of <1:10 or four-fold increase in post-vaccination HI titer for subjects with pre-vaccination HI titer of ≥1:10.

	NBP607-QIV (0.5mL) (N=434)	Agrippal (0.25mL) (N=219)
**[Influenza Strain: A/H3N2]**		
**Seroprotection rate, n(%)**	416(95.85)	207(94.52)
95% Confidence Interval	[93.98, 97.73]	[91.51, 97.53]
LCL ≥ 70%?	Yes	Yes
P-value [1]		0.4427 (c)
**Seroconversion rate, n(%)**	380(87.56)	196(89.50)
95% Confidence Interval	[84.45, 90.66]	[85.44, 93.56]
LCL ≥ 40%?	Yes	Yes
P-value [1]		0.4680 (c)
**GMR (post/pre-vaccination) (SD)**	12.00(2.94)	17.02(3.15)
95% Confidence Interval	[10.84, 13.29]	[14.61, 19.83]
LCL ≥ 2.5?	Yes	Yes
P-value [2]		0.0001

LCL = lower confidence limit, GMR = geometric mean ratio.
[1] Testing for difference between treatment groups (chi-square test (c) or Fisher's exact test (f)).
[2] Testing for difference between treatment groups (two sample t-test).
Note: Denominator of percentage is the number of subjects in the column. GMR are displayed as geometric mean (geometric standard deviation).
GMR = anti-logarithm[mean of logarithm(POST/PRE)] (PRE=original value before IP administration, POST= original value after IP administration)
Seroprotection rate: proportion of subjects whose post-vaccination HI titer increased to ≥1:40.
Seroconversion rate: proportion of subjects who post-vaccination HI titer of ≥1:40 for subjects with pre-vaccination HI titer of <1:10 or four-fold increase in post-vaccination HI titer for subjects with pre-vaccination HI titer of ≥1:10.

	NBP607-QIV (0.5mL) (N=434)	Agrippal (0.25mL) (N=219)
**[Influenza Strain: B/Victoria]**		
**Seroprotection rate, n(%)**	228(52.53)	94(42.92)
95% Confidence Interval	[47.84, 57.23]	[36.37, 49.48]
LCL ≥ 70%?	No	No
P-value [1]		0.0204 (c)
**Seroconversion rate, n(%)**	223(51.38)	94(42.92)
95% Confidence Interval	[46.68, 56.08]	[36.37, 49.48]
LCL ≥ 40%?	Yes	No
P-value [1]		0.0411 (c)
**GMR (post/pre-vaccination) (SD)**	4.89(2.82)	4.57(2.87)
95% Confidence Interval	[4.44, 5.39]	[3.97, 5.25]
LCL ≥ 2.5?	Yes	Yes
P-value [2]		0.4279

LCL = lower confidence limit, GMR = geometric mean ratio.
[1] Testing for difference between treatment groups (chi-square test (c) or Fisher's exact test (f)).
[2] Testing for difference between treatment groups (two sample t-test).
Note: Denominator of percentage is the number of subjects in the column. GMR are displayed as geometric mean (geometric standard deviation).
GMR = anti-logarithm[mean of logarithm(POST/PRE)] (PRE=original value before IP administration, POST= original value after IP administration)
Seroprotection rate: proportion of subjects whose post-vaccination HI titer increased to ≥1:40.
Seroconversion rate: proportion of subjects who post-vaccination HI titer of ≥1:40 for subjects with pre-vaccination HI titer of <1:10 or four-fold increase in post-vaccination HI titer for subjects with pre-vaccination HI titer of ≥1:10.

	NBP607-QIV (0.5mL) (N=434)	Agrippal (0.25mL) (N=219)
**[Influenza Strain: B/Yamagata]**		
**Seroprotection rate, n(%)**	259(59.68)	11(5.02)
95% Confidence Interval	[55.06, 64.29]	[2.13, 7.92]
LCL ≥ 70%?	No	No

ANCOVA = analysis of covariance, GMT = geometric mean titer, GMR = geometric mean ratio, SE = standard error, CI = confidence interval, SD = standard deviation.
[1] Testing for ratio between treatment groups (ANCOVA model with treatment group as a factor and log pre-GMT as a covariate).
Note: Denominator of percentage is the number of subjects in the column. GMT and GMR is displayed as geometric mean (geometric standard deviation).
Seroconversion rate: proportion of subjects who post-vaccination HI titer of ≥1:40 for subjects with pre-vaccination HI titer of <1:10 or four-fold increase in post-vaccination HI titer for subjects with pre-vaccination HI titer of ≥1:10.
Confidence interval of GMT is calculated using t-distribution and Confidence Interval of Seroconversion rate is calculated using Wald method.

Table S3 Immunogenicity Assessment by HI Assay (Secondary Endpoints_Consistency Among Countries – Per Protocol Set

Page 1 of 4

	Korea		Thailand		Malaysia	
	**NBP607-QIV (0.5mL) (N=61)**	**Agrippal (0.25mL) (N=32)**	**NBP607-QIV (0.5mL) (N=283)**	**Agrippal (0.25mL) (N=140)**	**NBP607-QIV (0.5mL) (N=90)**	**Agrippal (0.25mL) (N=47)**
**[Influenza Strain: A/H1N1]**						
**Seroconversion rate, n(%)**	47(77.05)	23(71.88)	249(87.99)	122(87.14)	84(93.33)	43(91.49)
Difference % (Agrippal - NBP607-QIV)		-5.17		-0.84		-1.84
Difference % < 10%?		Yes		Yes		Yes
**Adjusted post-vaccination GMT (SE)**	213.88(1.13)	208.68(1.18)	200.00(1.06)	215.73(1.08)	362.13(1.09)	458.77(1.11)
95% Confidence Interval	[167.72, 272.76]	[149.87, 290.56]	[180.01, 222.22]	[186.01, 250.19]	[307.26, 426.80]	[373.63, 563.32]
GMR (Agrippal/NBP607-QIV) [95% CI] [1]		0.98 [0.65, 1.46]		1.08 [0.90, 1.29]		1.27 [1.01, 1.58]
Ratio < 1.5?		Yes		Yes		Yes

ANCOVA = analysis of covariance, GMT = geometric mean titer, GMR = geometric mean ratio, SE = standard error, CI = confidence interval, SD = standard deviation.
[1] Testing for ratio between treatment groups (ANCOVA model with treatment group as a factor and log pre-GMT as a covariate).
Note: Denominator of percentage is the number of subjects in the column. GMT and GMR is displayed as geometric mean (geometric standard deviation).
Seroconversion rate: proportion of subjects who post-vaccination HI titer of ≥1:40 for subjects with pre-vaccination HI titer of <1:10 or four-fold increase in post-vaccination HI titer for subjects with pre-vaccination HI titer of ≥1:10.
Confidence interval of GMT is calculated using t-distribution and Confidence Interval of Seroconversion rate is calculated using Wald method.

	Korea		Thailand		Malaysia	
	**NBP607-QIV (0.5mL) (N=61)**	**Agrippal (0.25mL) (N=32)**	**NBP607-QIV (0.5mL) (N=283)**	**Agrippal (0.25mL) (N=140)**	**NBP607-QIV (0.5mL) (N=90)**	**Agrippal (0.25mL) (N=47)**
**[Influenza Strain: A/H3N2]**						
**Seroconversion rate, n(%)**	51(83.61)	26(81.25)	248(87.63)	124(88.57)	81(90.00)	46(97.87)
Difference % (Agrippal - NBP607-QIV)		-2.36		0.94		7.87
Difference % < 10%?		Yes		Yes		Yes
**Adjusted post-vaccination GMT (SE)**	361.17(1.10)	454.00(1.14)	175.96(1.06)	227.79(1.09)	226.28(1.09)	291.36(1.12)
95% Confidence Interval	[296.98, 439.24]	[348.83, 590.89]	[157.15, 197.03]	[193.64, 267.96]	[191.44, 267.47]	[231.17, 367.23]
GMR (Agrippal/NBP607-QIV) [95% CI] [1]		1.26 [0.92, 1.72]		1.29 [1.06, 1.58]		1.29 [0.97, 1.71]
Ratio < 1.5?		Yes		Yes		Yes

ANCOVA = analysis of covariance, GMT = geometric mean titer, GMR = geometric mean ratio, SE = standard error, CI = confidence interval, SD = standard deviation.
[1] Testing for ratio between treatment groups (ANCOVA model with treatment group as a factor and log pre-GMT as a covariate).
Note: Denominator of percentage is the number of subjects in the column. GMT and GMR is displayed as geometric mean (geometric standard deviation).
Seroconversion rate: proportion of subjects who post-vaccination HI titer of ≥1:40 for subjects with pre-vaccination HI titer of <1:10 or four-fold increase in post-vaccination HI titer for subjects with pre-vaccination HI titer of ≥1:10.
Confidence interval of GMT is calculated using t-distribution and Confidence Interval of Seroconversion rate is calculated using Wald method.

	Korea		Thailand		Malaysia	
	**NBP607-QIV (0.5mL) (N=61)**	**Agrippal (0.25mL) (N=32)**	**NBP607-QIV (0.5mL) (N=283)**	**Agrippal (0.25mL) (N=140)**	**NBP607-QIV (0.5mL) (N=90)**	**Agrippal (0.25mL) (N=47)**
**[Influenza Strain: B/Victoria]**						
**Seroconversion rate, n(%)**	27(44.26)	9(28.13)	143(50.53)	58(41.43)	53(58.89)	27(57.45)
Difference % (Agrippal - NBP607-QIV)		-16.14		-9.10		-1.44
Difference % < 10%?		Yes		Yes		Yes
**Adjusted post-vaccination GMT (SE)**	29.51(1.17)	15.69(1.24)	25.02(1.06)	22.97(1.08)	30.81(1.12)	41.81(1.16)
95% Confidence Interval	[21.65, 40.22]	[10.31, 23.87]	[22.37, 27.98]	[19.63, 26.88]	[24.77, 38.32]	[31.01, 56.38]
GMR (Agrippal/NBP607-QIV) [95% CI] [1]		0.53 [0.32, 0.89]		0.92 [0.76, 1.11]		1.36 [0.94, 1.96]
Ratio < 1.5?		Yes		Yes		Yes

ANCOVA = analysis of covariance, GMT = geometric mean titer, GMR = geometric mean ratio, SE = standard error, CI = confidence interval, SD = standard deviation.
[1] Testing for ratio between treatment groups (ANCOVA model with treatment group as a factor and log pre-GMT as a covariate).
Note: Denominator of percentage is the number of subjects in the column. GMT and GMR is displayed as geometric mean (geometric standard deviation).
Seroconversion rate: proportion of subjects who post-vaccination HI titer of ≥1:40 for subjects with pre-vaccination HI titer of <1:10 or four-fold increase in post-vaccination HI titer for subjects with pre-vaccination HI titer of ≥1:10.
Confidence interval of GMT is calculated using t-distribution and Confidence Interval of Seroconversion rate is calculated using Wald method.

	Korea		Thailand		Malaysia	
	**NBP607-QIV (0.5mL) (N=61)**	**Agrippal (0.25mL) (N=32)**	**NBP607-QIV (0.5mL) (N=283)**	**Agrippal (0.25mL) (N=140)**	**NBP607-QIV (0.5mL) (N=90)**	**Agrippal (0.25mL) (N=47)**
**[Influenza Strain: B/Yamagata]**						
**Seroconversion rate, n(%)**	34(55.74)	2(6.25)	156(55.12)	2(1.43)	61(67.78)	0(0.00)
95% Confidence Interval	[43.27, 68.20]	[0.00, 14.64]	[49.33, 60.92]	[0.00, 3.39]	[58.12, 77.43]	[0.00, 0.00]
Rate > 40%?	Yes	No	Yes	No	Yes	No
**Pre-vaccination GMT (SD)**	5.60(1.50)	5.00(1.00)	6.21(1.82)	5.86(1.55)	5.99(1.64)	6.62(1.84)
95% Confidence Interval	[5.05, 6.21]	[5.00, 5.00]	[5.79, 6.66]	[5.44, 6.30]	[5.40, 6.64]	[5.54, 7.91]
**Post-vaccination GMT (SD)**	25.68(3.68)	6.35(1.91)	38.46(3.04)	6.68(1.85)	42.53(2.92)	7.23(1.93)
95% Confidence Interval	[18.39, 35.85]	[5.02, 8.02]	[33.76, 43.81]	[6.03, 7.40]	[33.99, 53.22]	[5.96, 8.78]
**GMR (post/pre-vaccination) (SD)**	4.58(3.32)	1.27(1.91)	6.19(2.73)	1.14(1.43)	7.10(2.56)	1.09(1.32)
95% Confidence Interval	[3.37, 6.23]	[1.00, 1.60]	[5.51, 6.96]	[1.07, 1.21]	[5.83, 8.64]	[1.01, 1.18]
GMR > 2.5?	Yes	No	Yes	No	Yes	No

AEs = adverse events.
[1] Testing for difference between treatment groups (chi-square test (c) or Fisher's exact test (f)).
Note: Denominator of percentage is the number of subjects in each group.
Confidence interval is calculated using Wald or Clopper-Pearson method according to statistical test.
Severity, relationship, medication and outcome are displayed as 'number of cases(percentage of cases)' and others are displayed as 'number of subjects(percentage of subjects) [number of cases]'.
Percentages of severity, relationship, medication and outcome are based on the total number of occurrences in each treatment group.

Table S4 Overall Adverse Events – Safety Set

	NBP607-QIV (0.5mL) (N=449)	Agrippal (0.25mL) (N=226)	Total (N=675)
**Total AEs, n(%) [case]**	314(69.93) [927]	164(72.57) [512]	478(70.81) [1439]
95% Confidence Interval	[65.69, 74.17]	[66.75, 78.38]	[67.39, 74.24]
P-value [1]			0.4776 (c)
**Severity, case(%)**	927	512	1439
Mild (Grade 1)	737(79.50)	416(81.25)	1153(80.13)
Moderate (Grade 2)	159(17.15)	70(13.67)	229(15.91)
Severe (Grade 3)	30(3.24)	26(5.08)	56(3.89)
Potentially life-threatening (Grade 4)	1(0.11)	0(0.00)	1(0.07)
**Relationship to IP, case(%)**	927	512	1439
Not-related	388(41.86)	223(43.55)	611(42.46)
Related	539(58.14)	289(56.45)	828(57.54)
**Medication, case(%)**	927	512	1439
Yes	334(36.03)	184(35.94)	518(36.00)
No	593(63.97)	328(64.06)	921(64.00)
**Outcome, case(%)**	927	512	1439
Recovered/Resolved	921(99.35)	509(99.41)	1430(99.37)
Recovering/Resolving	2(0.22)	2(0.39)	4(0.28)
Not recovered/Not resolved	4(0.43)	1(0.20)	5(0.35)
Recovered/Resolved with sequelae	0(0.00)	0(0.00)	0(0.00)
Fatal	0(0.00)	0(0.00)	0(0.00)
Unknown	0(0.00)	0(0.00)	0(0.00)

AEs = adverse events.
[1] Testing for difference between treatment groups (chi-square test (c) or Fisher's exact test (f)).
Note: Denominator of percentage is the number of subjects in each group.
Confidence interval is calculated using Wald or Clopper-Pearson method according to statistical test.
Severity, relationship, medication and outcome are displayed as 'number of cases(percentage of cases)' and others are displayed as 'number of subjects(percentage of subjects) [number of cases]'.
Percentages of severity, relationship, medication and outcome are based on the total number of occurrences in each treatment group.

	NBP607-QIV (0.5mL) (N=449)	Agrippal (0.25mL) (N=226)	Total (N=675)
**Subjects with Serious AEs, n(%) [case]**	8(1.78) [8]	9(3.98) [11]	17(2.52) [19]
95% Confidence Interval	[0.56, 3.01]	[1.43, 6.53]	[1.34, 3.70]
P-value [1]			0.0851 (c)
**Subjects with Solicited Local AEs, n(%) [case]**	187(41.65) [336]	96(42.48) [193]	283(41.93) [529]
95% Confidence Interval	[37.09, 46.21]	[36.03, 48.92]	[38.20, 45.65]
P-value [1]			0.8366 (c)
**Subjects with Solicited Systemic AEs, n(%) [case]**	136(30.29) [246]	67(29.65) [123]	203(30.07) [369]
95% Confidence Interval	[26.04, 34.54]	[23.69, 35.60]	[26.61, 33.53]
P-value [1]			0.8634 (c)
**Subjects with Unsolicited AEs, n(%) [case]**	202(44.99) [345]	106(46.90) [196]	308(45.63) [541]
95% Confidence Interval	[40.39, 49.59]	[40.40, 53.41]	[41.87, 49.39]
P-value [1]			0.6376 (c)

AEs = adverse events.
[1] Testing for difference between treatment groups (chi-square test (c) or Fisher's exact test (f)).
Note: Denominator of percentage is the number of subjects in each group.
Confidence interval is calculated using Wald or Clopper-Pearson method according to statistical test.
Severity, relationship, medication and outcome are displayed as 'number of cases(percentage of cases)' and others are displayed as 'number of subjects(percentage of subjects) [number of cases]'.
Percentages of severity, relationship, medication and outcome are based on the total number of occurrences in each treatment group.

Table S5 Solicited Local Adverse Events – Safety Set

	NBP607-QIV (0.5mL) (N=449)	Agrippal (0.25mL) (N=226)	Total (N=675)
**Total Solicited Local AEs, n(%) [case]**	187(41.65) [336]	96(42.48) [193]	283(41.93) [529]
95% Confidence Interval	[37.09, 46.21]	[36.03, 48.92]	[38.20, 45.65]
P-value [1]			0.8366 (c)
**Severity, case(%)**	336	193	529
Mild (Grade 1)	310(92.26)	184(95.34)	494(93.38)
Moderate (Grade 2)	20(5.95)	9(4.66)	29(5.48)
Severe (Grade 3)	6(1.79)	0(0.00)	6(1.13)
Potentially life-threatening (Grade 4)	0(0.00)	0(0.00)	0(0.00)
**Relationship to IP, case(%)**	336	193	529
Not-related	20(5.95)	5(2.59)	25(4.73)
Related	316(94.05)	188(97.41)	504(95.27)
**Medication, case(%)**	336	193	529
Yes	1(0.30)	0(0.00)	1(0.19)
No	335(99.70)	193(100.00)	528(99.81)
**Outcome, case(%)**	336	193	529
Recovered/Resolved	336(100.00)	193(100.00)	529(100.00)
Recovering/Resolving	0(0.00)	0(0.00)	0(0.00)
Not recovered/Not resolved	0(0.00)	0(0.00)	0(0.00)
Recovered/Resolved with sequelae	0(0.00)	0(0.00)	0(0.00)
Fatal	0(0.00)	0(0.00)	0(0.00)
Unknown	0(0.00)	0(0.00)	0(0.00)

AEs = adverse events.
[1] Testing for difference between treatment groups (chi-square test (c) or Fisher's exact test (f)).
Note: Denominator of percentage is the number of subjects in each group.
Confidence interval is calculated using Wald or Clopper-Pearson method according to statistical test.
Severity, relationship, medication and outcome are displayed as 'number of cases(percentage of cases)' and others are displayed as 'number of subjects(percentage of subjects) [number of cases]'.
Percentages of severity, relationship, medication and outcome are based on the total number of occurrences in each treatment group.

	NBP607-QIV (0.5mL) (N=449)	Agrippal (0.25mL) (N=226)	Total (N=675)
**Subjects with Serious Solicited Local AEs, n(%) [case]**	0(0.00) [0]	0(0.00) [0]	0(0.00) [0]
Exact 95% Confidence Interval	[0.00, 0.82]	[0.00, 1.62]	[0.00, 0.55]
P-value [1]			-

AEs = adverse events.
[1] Testing for difference between treatment groups (chi-square test (c) or Fisher's exact test (f)).
Note: Denominator of percentage is the number of subjects in each group.
Confidence interval is calculated using Wald or Clopper-Pearson method according to statistical test.
Severity, relationship, medication and outcome are displayed as 'number of cases(percentage of cases)' and others are displayed as 'number of subjects(percentage of subjects) [number of cases]'.
Percentages of severity, relationship, medication and outcome are based on the total number of occurrences in each treatment group.

Table S6 Solicited Systemic Adverse Events – Safety Set

	NBP607-QIV (0.5mL) (N=449)	Agrippal (0.25mL) (N=226)	Total (N=675)
**Total Solicited Systemic AEs, n(%) [case]**	136(30.29) [246]	67(29.65) [123]	203(30.07) [369]
95% Confidence Interval	[26.04, 34.54]	[23.69, 35.60]	[26.61, 33.53]
P-value [1]			0.8634 (c)
**Severity, case(%)**	246	123	369
Mild (Grade 1)	182(73.98)	99(80.49)	281(76.15)
Moderate (Grade 2)	48(19.51)	10(8.13)	58(15.72)
Severe (Grade 3)	15(6.10)	14(11.38)	29(7.86)
Potentially life-threatening (Grade 4)	1(0.41)	0(0.00)	1(0.27)
**Relationship to IP, case(%)**	246	123	369
Not-related	57(23.17)	32(26.02)	89(24.12)
Related	189(76.83)	91(73.98)	280(75.88)
**Medication, case(%)**	246	123	369
Yes	50(20.33)	27(21.95)	77(20.87)
No	196(79.67)	96(78.05)	292(79.13)
**Outcome, case(%)**	246	123	369
Recovered/Resolved	245(99.59)	123(100.00)	368(99.73)
Recovering/Resolving	1(0.41)	0(0.00)	1(0.27)
Not recovered/Not resolved	0(0.00)	0(0.00)	0(0.00)
Recovered/Resolved with sequelae	0(0.00)	0(0.00)	0(0.00)
Fatal	0(0.00)	0(0.00)	0(0.00)
Unknown	0(0.00)	0(0.00)	0(0.00)

AEs = adverse events.
[1] Testing for difference between treatment groups (chi-square test (c) or Fisher's exact test (f)).
Note: Denominator of percentage is the number of subjects in each group.
Confidence interval is calculated using Wald or Clopper-Pearson method according to statistical test.
Severity, relationship, medication and outcome are displayed as 'number of cases(percentage of cases)' and others are displayed as 'number of subjects(percentage of subjects) [number of cases]'.
Percentages of severity, relationship, medication and outcome are based on the total number of occurrences in each treatment group.

	NBP607-QIV (0.5mL) (N=449)	Agrippal (0.25mL) (N=226)	Total (N=675)
**Subjects with Serious Solicited Systemic AEs, n(%) [case]**	0(0.00) [0]	0(0.00) [0]	0(0.00) [0]
Exact 95% Confidence Interval	[0.00, 0.82]	[0.00, 1.62]	[0.00, 0.55]
P-value [1]			-

AEs = adverse events.
[1] Testing for difference between treatment groups (chi-square test (c) or Fisher's exact test (f)).
Note: Denominator of percentage is the number of subjects in each group.
Confidence interval is calculated using Wald or Clopper-Pearson method according to statistical test.
Severity, relationship, medication and outcome are displayed as 'number of cases(percentage of cases)' and others are displayed as 'number of subjects(percentage of subjects) [number of cases]'.
Percentages of severity, relationship, medication and outcome are based on the total number of occurrences in each treatment group.

Table S7 Unsolicited Adverse Events – Safety Set

	NBP607-QIV (0.5mL) (N=449)	Agrippal (0.25mL) (N=226)	Total (N=675)
**Total Unsolicited AEs, n(%) [case]**	202(44.99) [345]	106(46.90) [196]	308(45.63) [541]
95% Confidence Interval	[40.39, 49.59]	[40.40, 53.41]	[41.87, 49.39]
P-value [1]			0.6376 (c)
**Severity, case(%)**	345	196	541
Mild (Grade 1)	245(71.01)	133(67.86)	378(69.87)
Moderate (Grade 2)	91(26.38)	51(26.02)	142(26.25)
Severe (Grade 3)	9(2.61)	12(6.12)	21(3.88)
Potentially life-threatening (Grade 4)	0(0.00)	0(0.00)	0(0.00)
**Relationship to IP, case(%)**	345	196	541
Not-related	311(90.14)	186(94.90)	497(91.87)
Related	34(9.86)	10(5.10)	44(8.13)
**Medication, case(%)**	345	196	541
Yes	283(82.03)	157(80.10)	440(81.33)
No	62(17.97)	39(19.90)	101(18.67)
**Outcome, case(%)**	345	196	541
Recovered/Resolved	340(98.55)	193(98.47)	533(98.52)
Recovering/Resolving	1(0.29)	2(1.02)	3(0.55)
Not recovered/Not resolved	4(1.16)	1(0.51)	5(0.92)
Recovered/Resolved with sequelae	0(0.00)	0(0.00)	0(0.00)
Fatal	0(0.00)	0(0.00)	0(0.00)
Unknown	0(0.00)	0(0.00)	0(0.00)

AEs = adverse events.
[1] Testing for difference between treatment groups (chi-square test (c) or Fisher's exact test (f)).
Note: Denominator of percentage is the number of subjects in each group.
Confidence interval is calculated using Wald or Clopper-Pearson method according to statistical test.
Severity, relationship, medication and outcome are displayed as 'number of cases(percentage of cases)' and others are displayed as 'number of subjects(percentage of subjects) [number of cases]'.
Percentages of severity, relationship, medication and outcome are based on the total number of occurrences in each treatment group.

	NBP607-QIV (0.5mL) (N=449)	Agrippal (0.25mL) (N=226)	Total (N=675)
**Subjects with Serious Unsolicited AEs, n(%) [case]**	8(1.78) [8]	9(3.98) [11]	17(2.52) [19]
95% Confidence Interval	[0.56, 3.01]	[1.43, 6.53]	[1.34, 3.70]
P-value [1]			0.0851 (c)

AEs = adverse events.
[1] Testing for difference between treatment groups (chi-square test (c) or Fisher's exact test (f)).
Note: Denominator of percentage is the number of subjects in each group.
Confidence interval is calculated using Wald or Clopper-Pearson method according to statistical test.
Severity, relationship, medication and outcome are displayed as 'number of cases(percentage of cases)' and others are displayed as 'number of subjects(percentage of subjects) [number of cases]'.
Percentages of severity, relationship, medication and outcome are based on the total number of occurrences in each treatment group.

Table S8 Serious Adverse Events by System Organ Class / Preferred Term – Safety Set

System Organ Class<br>Preferred Term	NBP607-QIV<br>(0.5mL)<br>(N=449)	Agrippal<br>(0.25mL)<br>(N=226)	Total<br>(N=675)	P-value [1]
**Subjects with Serious Unsolicited AEs, n(%) [case]**	8(1.78) [8]	9(3.98) [11]	17(2.52) [19]	0.0851 (c)
**Infections and infestations**	7(1.56) [7]	8(3.54) [8]	15(2.22) [15]	0.0994 (c)
Pneumonia	3(0.67) [3]	3(1.33) [3]	6(0.89) [6]	
Bronchitis	1(0.22) [1]	0(0.00) [0]	1(0.15) [1]	
Gastroenteritis	1(0.22) [1]	0(0.00) [0]	1(0.15) [1]	
Hand-foot-and-mouth disease	0(0.00) [0]	1(0.44) [1]	1(0.15) [1]	
Herpangina	0(0.00) [0]	1(0.44) [1]	1(0.15) [1]	
Lymphadenitis bacterial	0(0.00) [0]	1(0.44) [1]	1(0.15) [1]	
Pharyngitis	1(0.22) [1]	0(0.00) [0]	1(0.15) [1]	
Pharyngotonsillitis	0(0.00) [0]	1(0.44) [1]	1(0.15) [1]	
Pneumonia mycoplasmal	1(0.22) [1]	0(0.00) [0]	1(0.15) [1]	
Pneumonia viral	0(0.00) [0]	1(0.44) [1]	1(0.15) [1]	
**Respiratory, thoracic and mediastinal disorders**	1(0.22) [1]	3(1.33) [3]	4(0.59) [4]	0.1118 (f)
Wheezing	1(0.22) [1]	1(0.44) [1]	2(0.30) [2]	
Asthma	0(0.00) [0]	1(0.44) [1]	1(0.15) [1]	
Bronchial hyperreactivity	0(0.00) [0]	1(0.44) [1]	1(0.15) [1]	

AEs = adverse events.
[1] Testing for difference between treatment groups (chi-square test (c) or Fisher's exact test (f)).
Note: Denominator of percentage is the number of subjects in each group.
Confidence interval is calculated using Wald or Clopper-Pearson method according to statistical test.
Severity, relationship, medication and outcome are displayed as 'number of cases(percentage of cases)' and others are displayed as 'number of subjects(percentage of subjects) [number of cases]'.
Percentages of severity, relationship, medication and outcome are based on the total number of occurrences in each treatment group.

II. Supplementary Figures

Figure S1 Ratio of Adjusted post-vaccination GMTs ($GMT_{Agrippal}/GMT_{NBP607\text{-}QIV}$) by HI Assay [A/H1N1, A/H3N2, B/Victoria] – Per Protocol Set

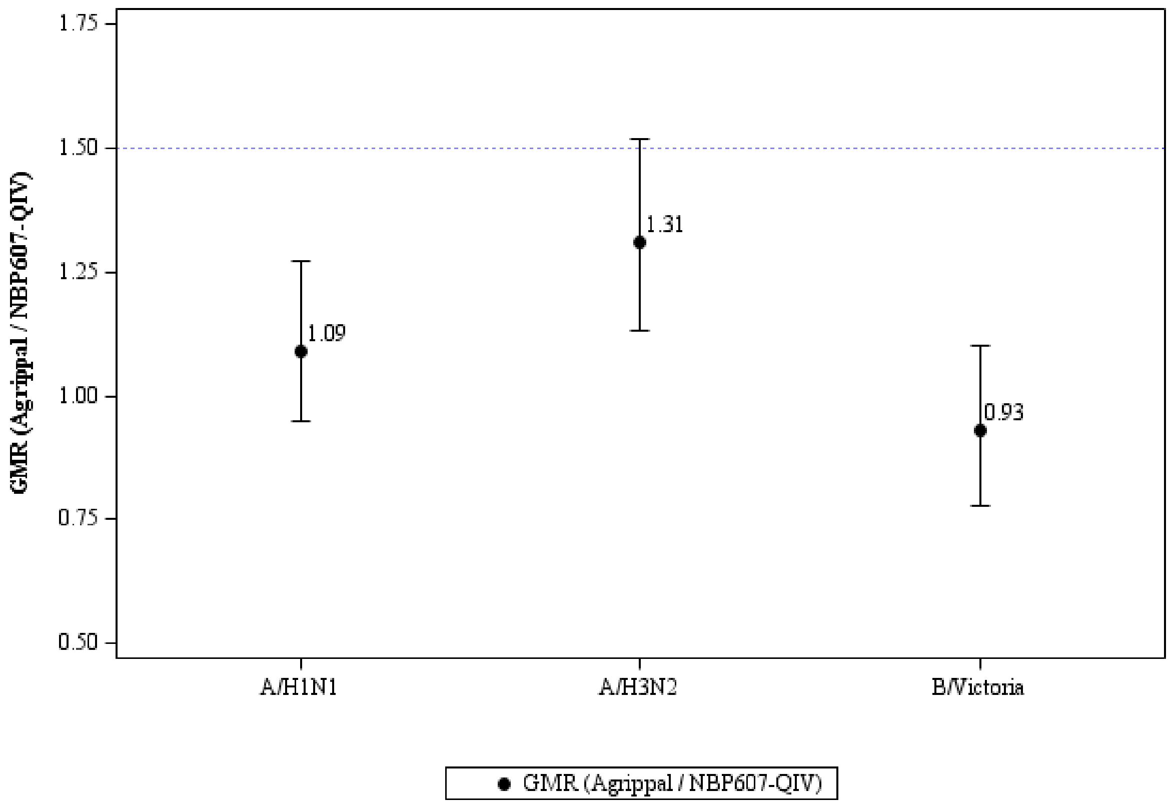

* GMR = geometric mean ratio.
* Figure represents Geometric LS Mean ratio (95% confidence interval) between treatment groups.

Figure S2 Difference of Seroconversion Rates between Treatment Groups by HI Assay [A/H1N1, A/H3N2, B/Victoria] – Per Protocol Set

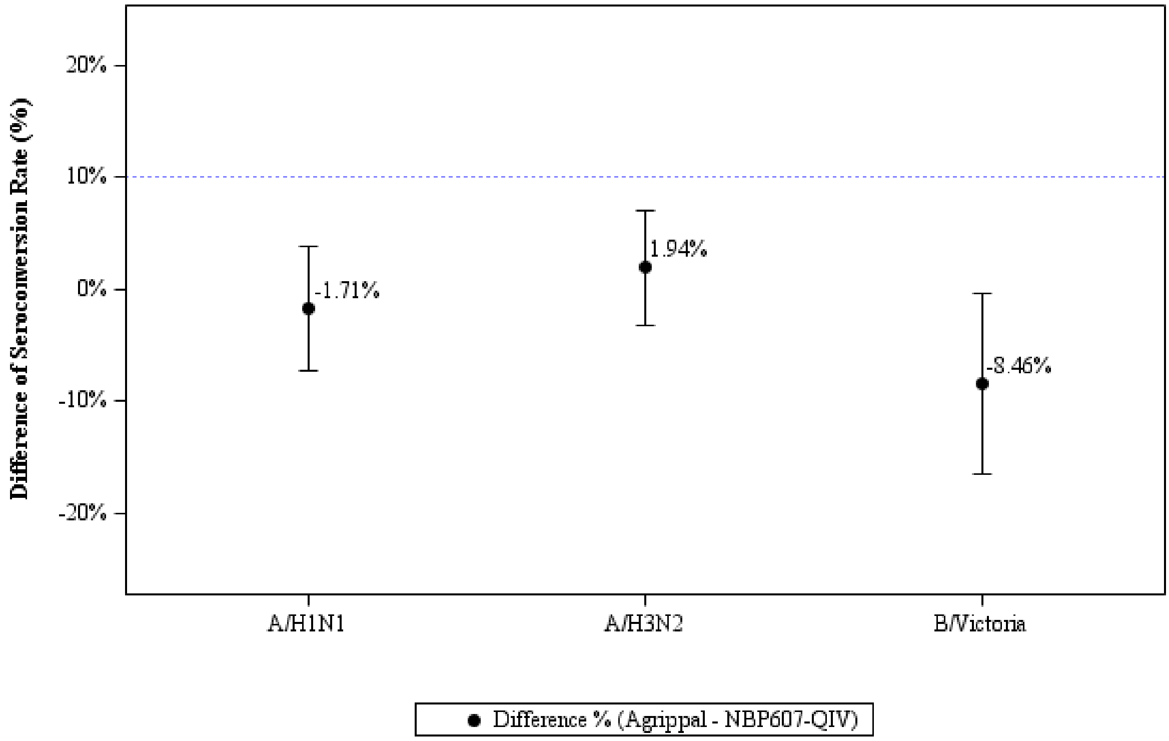

* Seroconversion rate: proportion of subjects who post-vaccination HI titer of ≥1:40 for subjects with pre-vaccination HI titer of <1:10 or four-fold increase in post-vaccination HI titer for subjects with pre-vaccination HI titer of ≥1:10.
* Figure represents Seroconversion rate difference (95% confidence interval) between treatment groups.

Figure S3 GMR (post/pre-vaccination of NBP607-QIV) by HI Assay [B/Yamagata] – Per Protocol Set

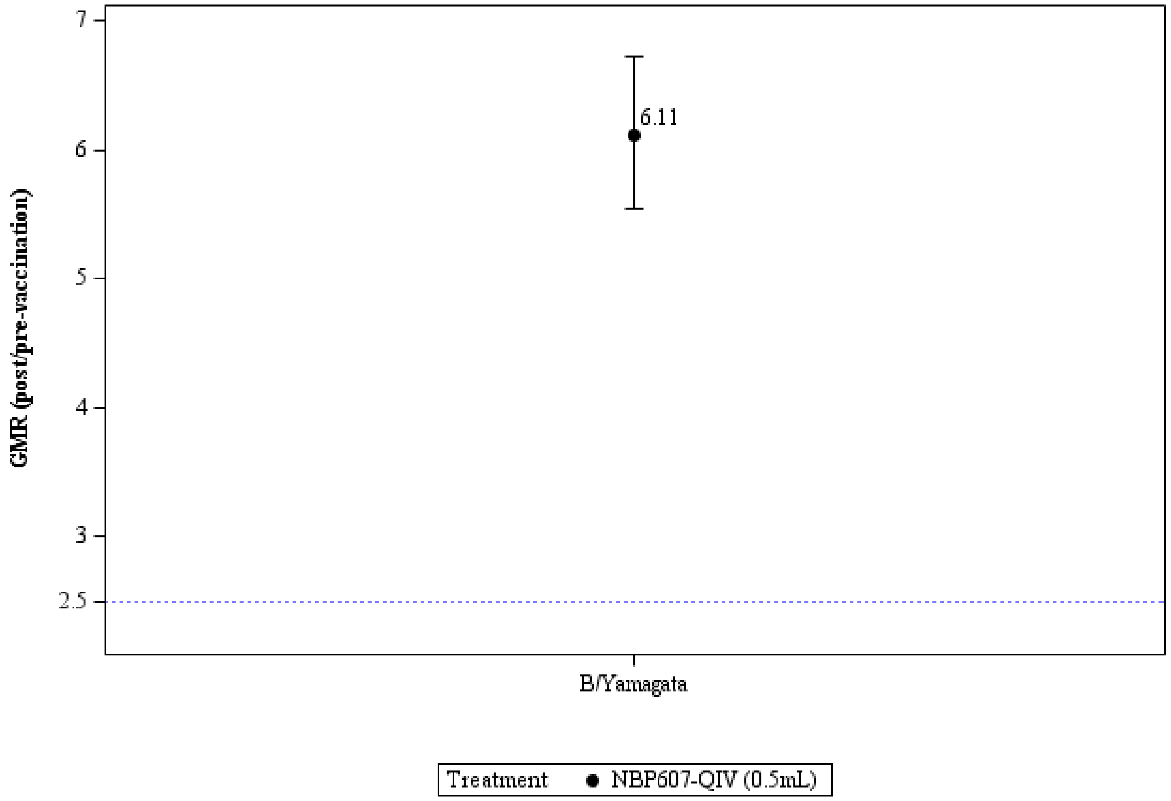

* GMR = geometric mean ratio.
* Figure represents GMR (95% confidence interval) of post-vaccination versus pre-vaccination..

Figure S4 Seroconversion Rate post-vaccination of NBP607-QIV by HI Assay [B/Yamagata] – Per Protocol Set

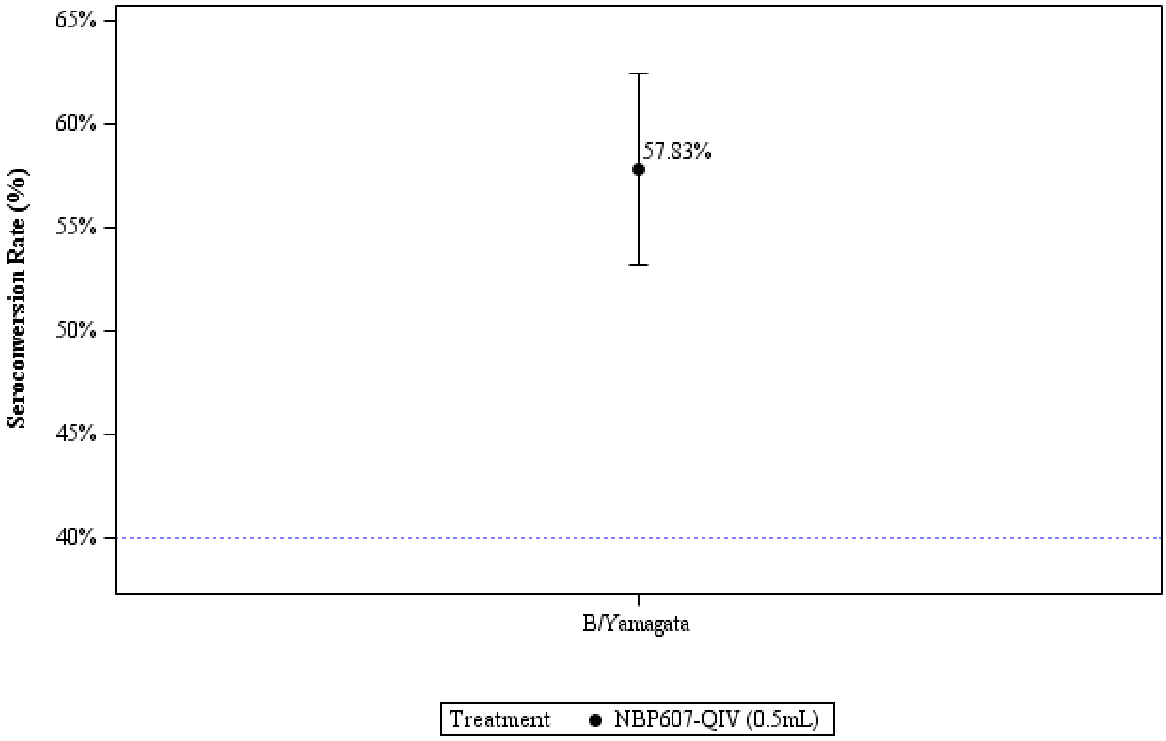

* Seroconversion rate: proportion of subjects who post-vaccination HI titer of ≥1:40 for subjects with pre-vaccination HI titer of <1:10 or four-fold increase in post-vaccination HI titer for subjects with pre-vaccination HI titer of ≥1:10.
* Figure represents Seroconversion rate (95% confidence interval).

Figure S5 Seroprotection Rates post-vaccination of NBP607-QIV/Agrippal by HI Assay [A/H1N1, A/H3N2, B/Victoria, B/Yamagata] – Per Protocol Set

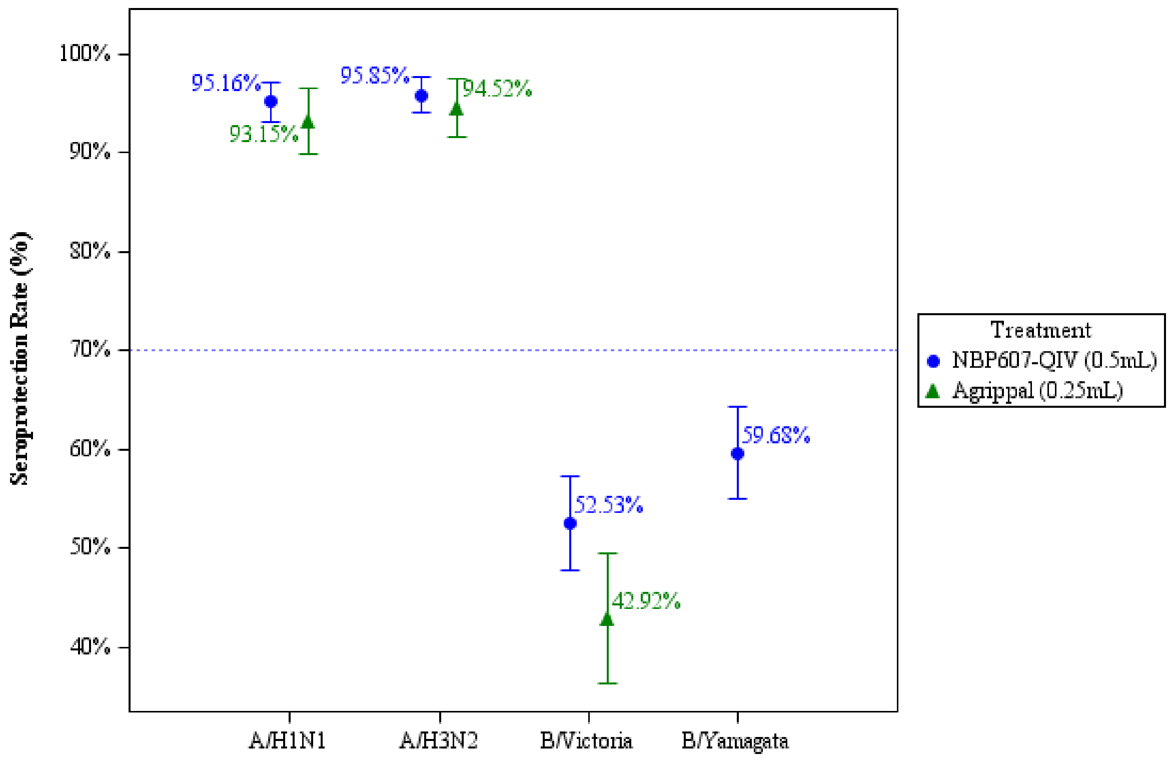

* Seroprotection rate: proportion of subjects whose post-vaccination HI titer increased to ≥1:40.
* Figure represents Seroprotection rate (95% confidence interval).

Figure S6 Seroconversion Rates post-vaccination of NBP607-QIV/Agrippal by HI Assay [A/H1N1, A/H3N2, B/Victoria] – Per Protocol Set

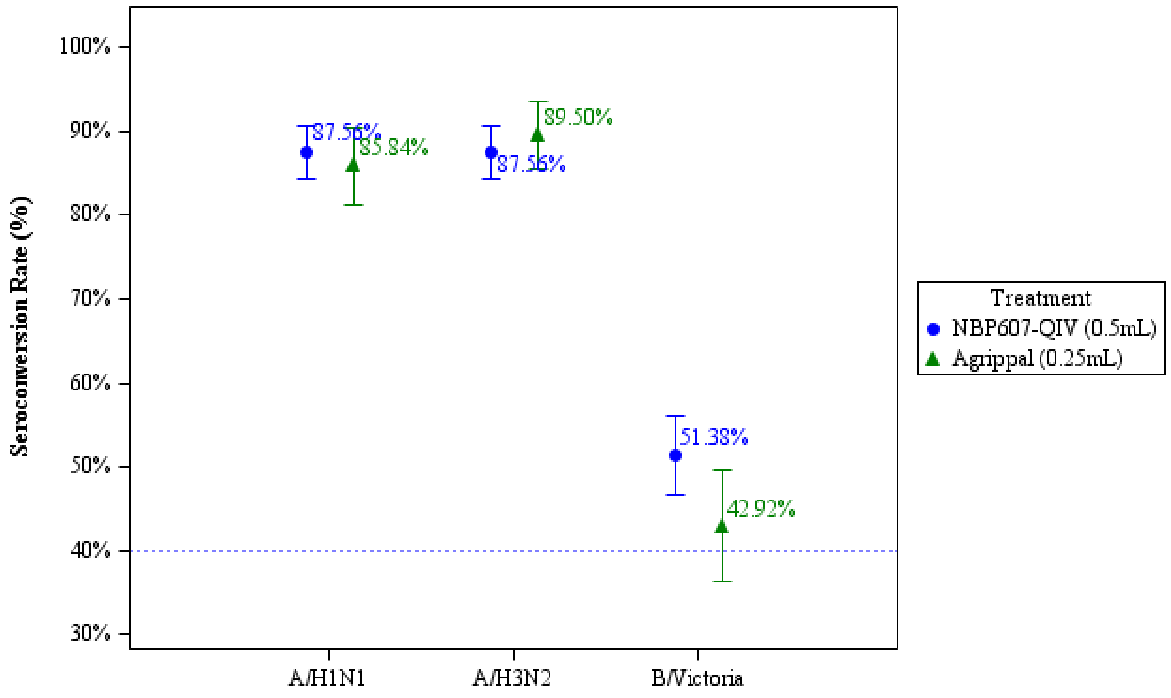

* Seroconversion rate: proportion of subjects who post-vaccination HI titer of ≥1:40 for subjects with pre-vaccination HI titer of <1:10 or four-fold increase in post-vaccination HI titer for subjects with pre-vaccination HI titer of ≥1:10.

* Figure represents Seroconversion rate (95% confidence interval).

Figure S7 GMRs (post/pre-vaccination of NBP607-QIV/Agrippal) by HI Assay [A/H1N1, A/H3N3, B/Victoria] – Per Protocol Set

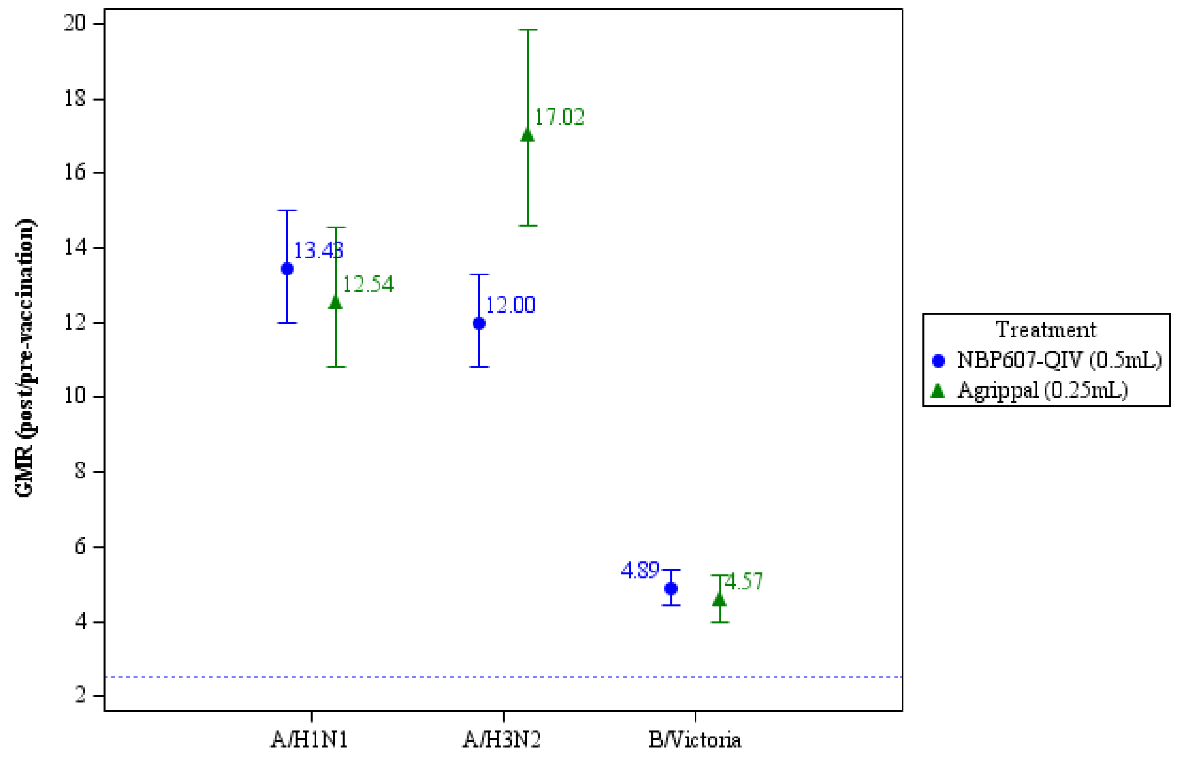

* GMR = geometric mean ratio.
* Figure represents GMR (95% confidence interval) of post-vaccination versus pre-vaccination..

Figure S8 Pre- and post-vaccination HI titre distributions [A/H1N1 – Per Protocol Set

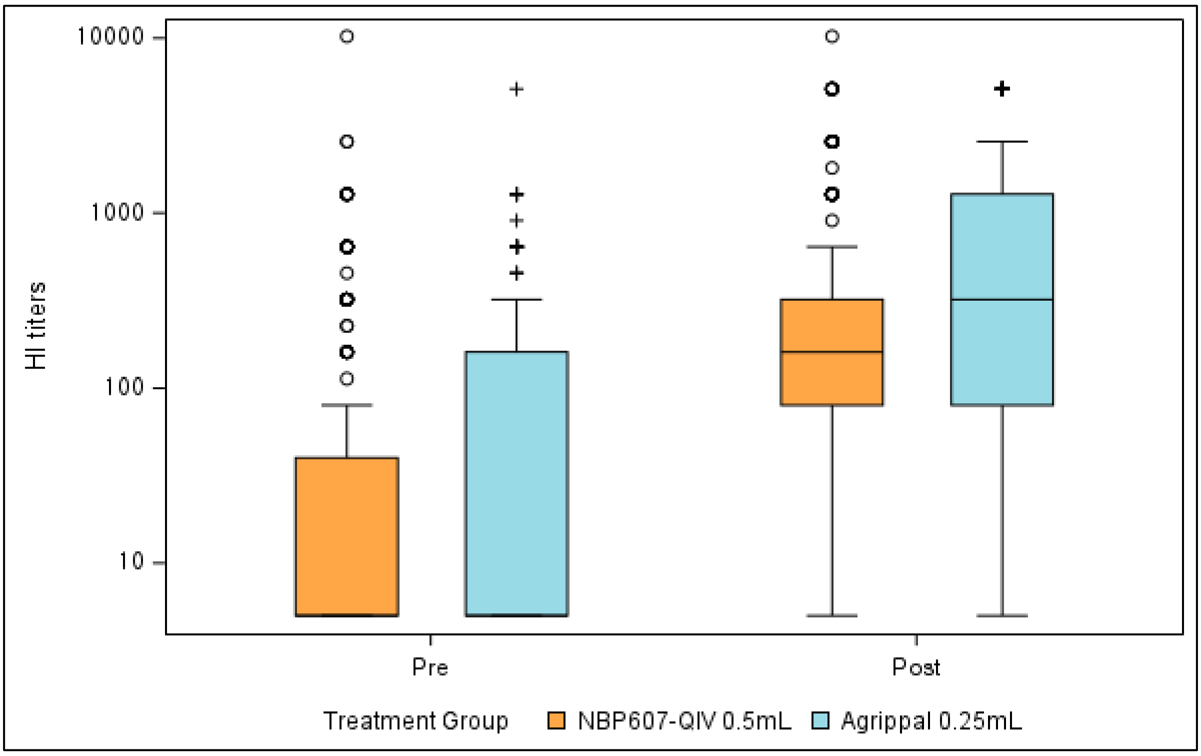

Figure S9 Pre- and post-vaccination HI titre distributions [A/H3N2] – Per Protocol Set

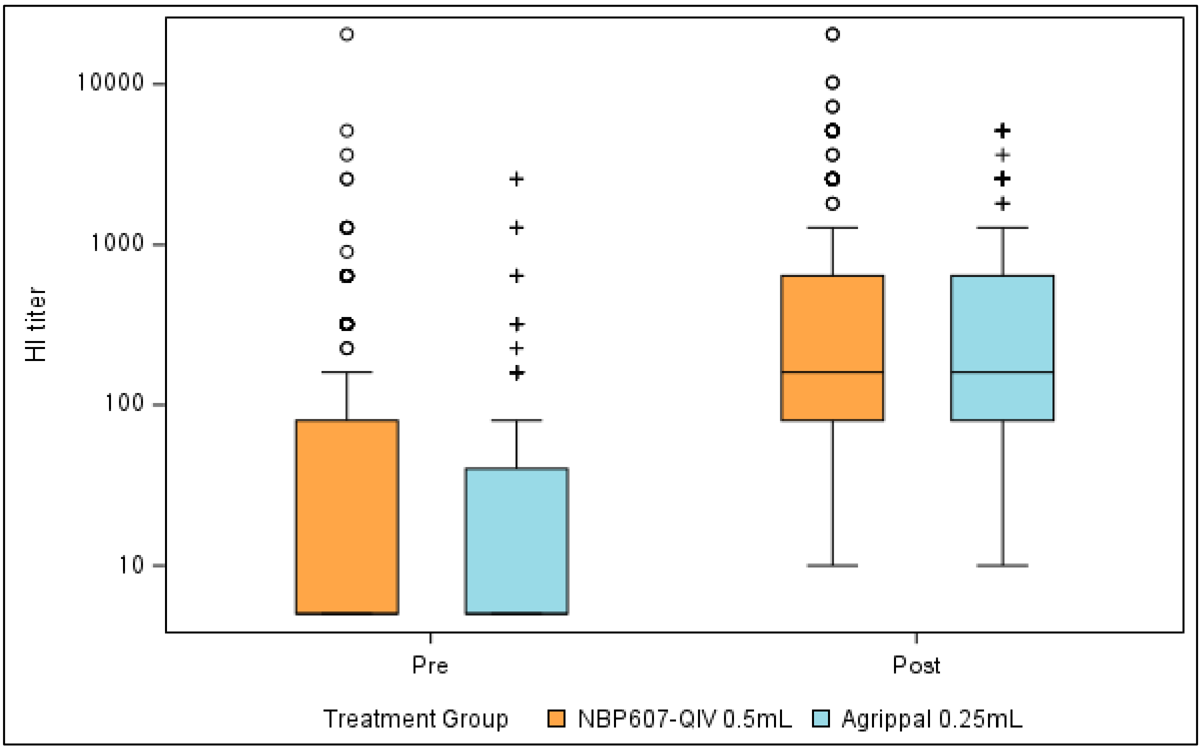

Figure S10 Pre- and post-vaccination HI titre distributions [B/Victoria] – Per Protocol Set

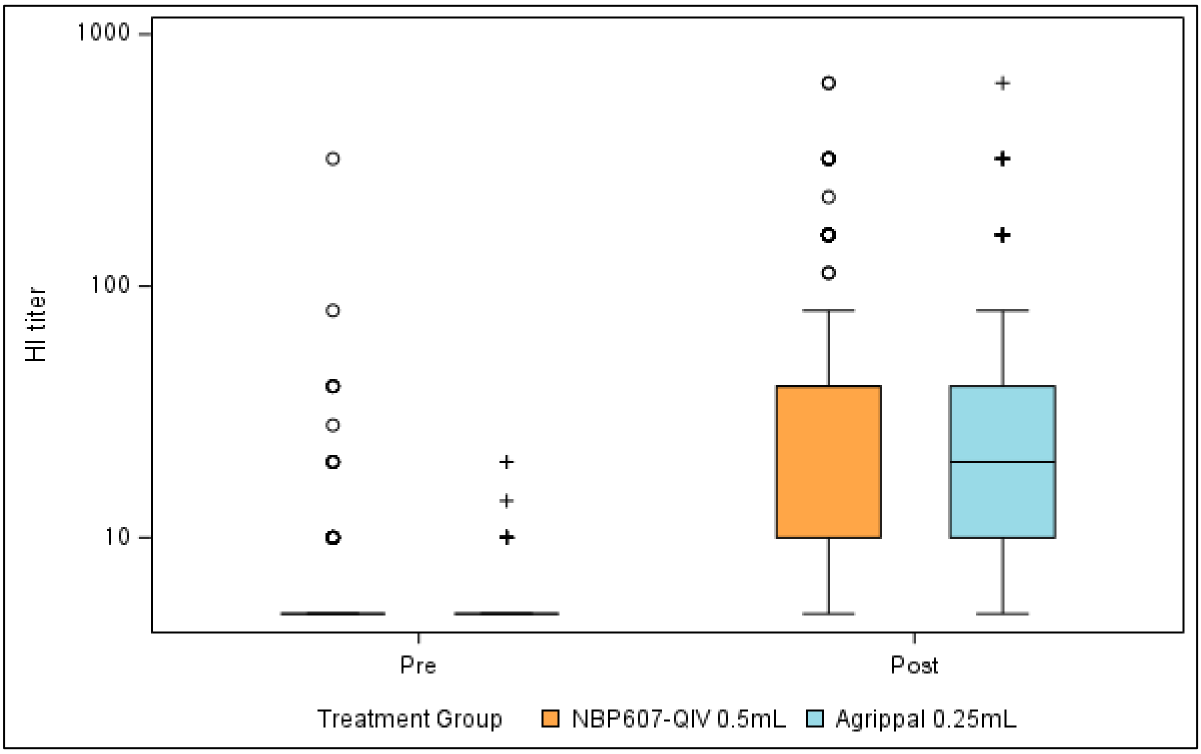

Figure S11 Pre- and post-vaccination HI titre distributions [B/Yamagata] – Per Protocol Set

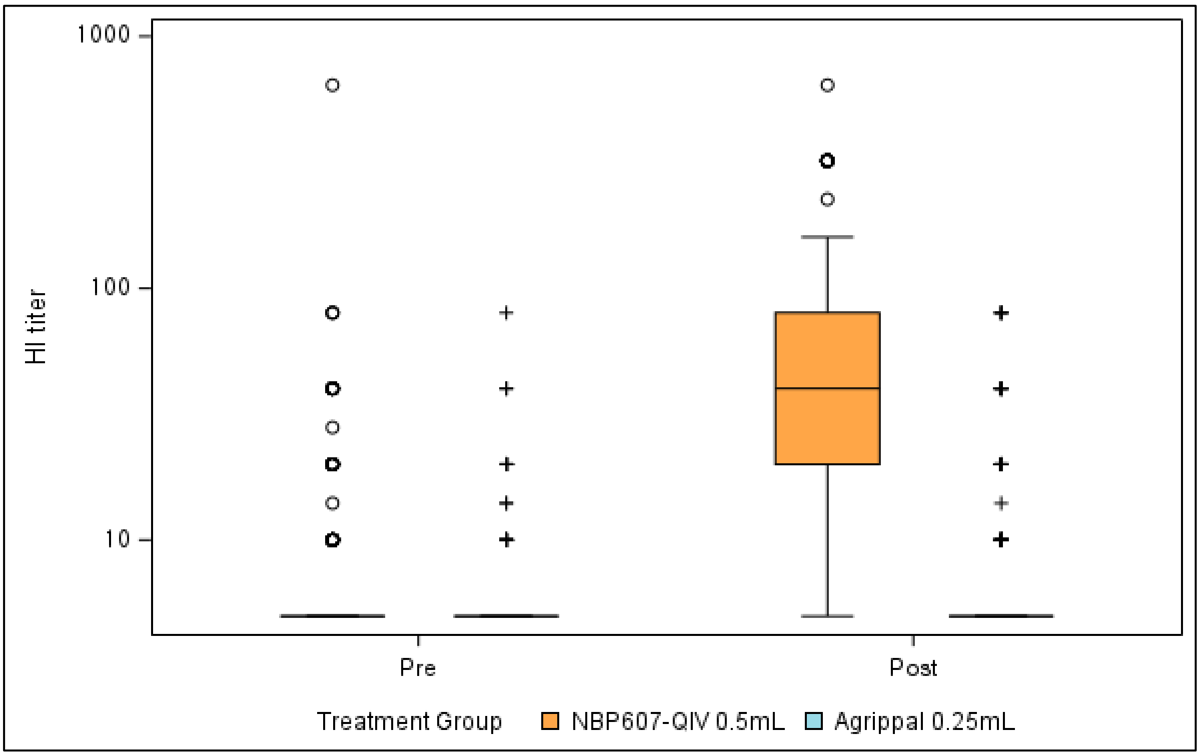

Figure S12 Age-stratified distributions of HI titres post-vaccination [A/H1N1] – Per Protocol Set

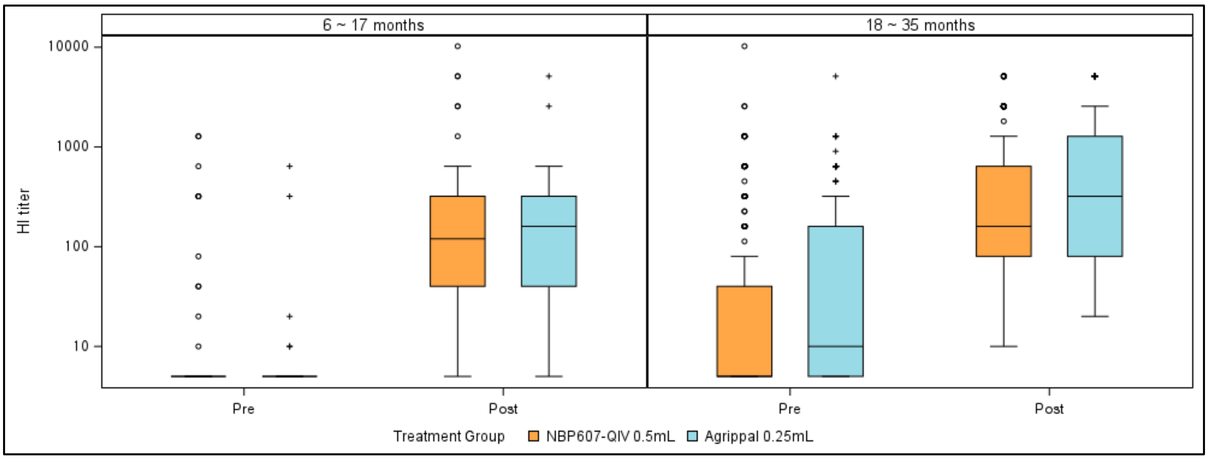

Figure S13 Age-stratified distributions of HI titres post-vaccination [A/H3N2] – Per Protocol Set

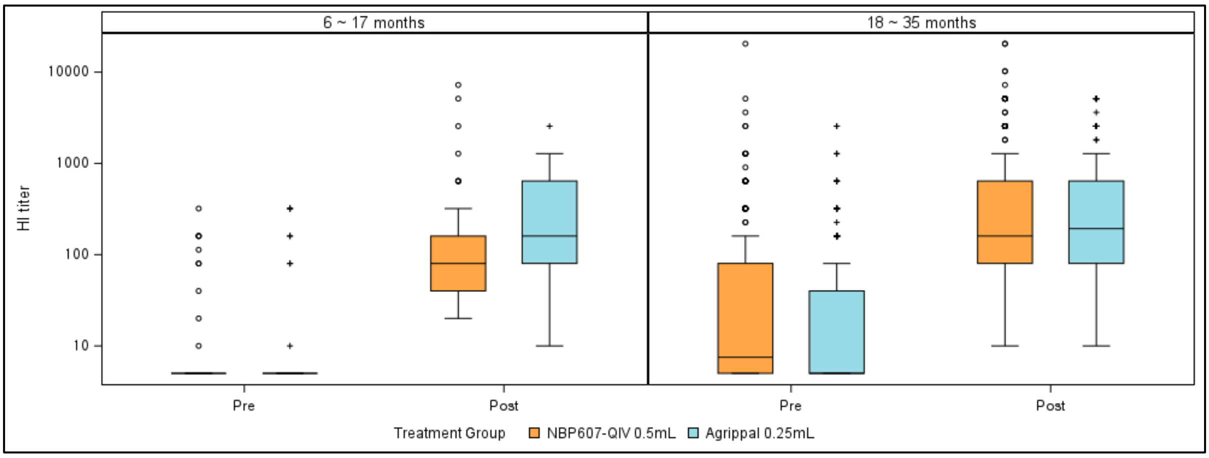

Figure S14 Age-stratified distributions of HI titres post-vaccination [B/Victoria] – Per Protocol Set

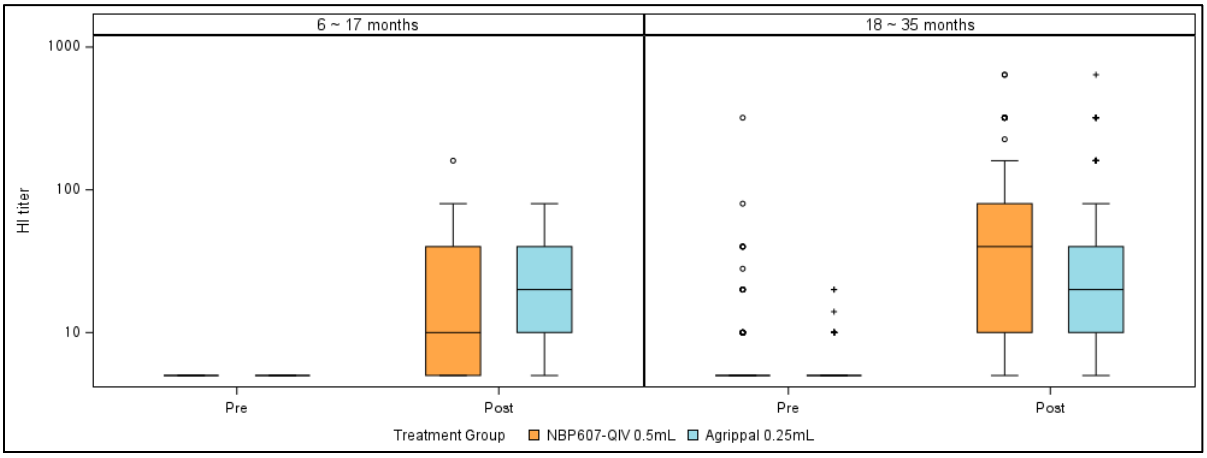

Figure S15 Age-stratified distributions of HI titres post-vaccination [B/Yamagata] – Per Protocol Set

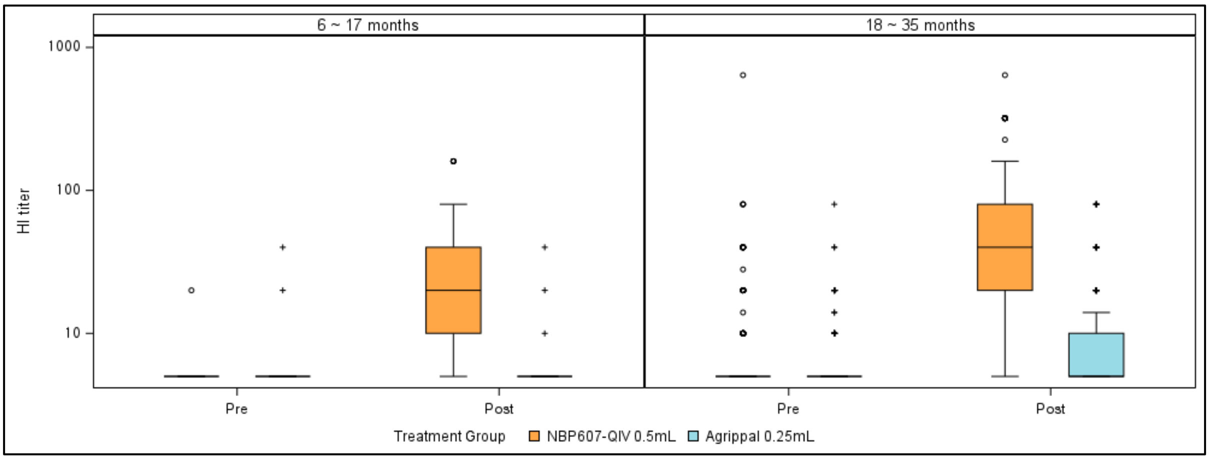

Supplement: Supplementary file 1 [file vaccines-14-00341-s001.zip › vaccines-4192438-supplementary.pdf]
